# Truncated S-MGBs: towards a parasite-specific and low aggregation chemotype†

**DOI:** 10.1039/d1md00110h

**Published:** 2021-07-06

**Authors:** Daniel P. Brooke, Leah M. C. McGee, Federica Giordani, Jasmine M. Cross, Abedawn I. Khalaf, Craig Irving, Kirsten Gillingwater, Craig D. Shaw, Katharine C. Carter, Michael P. Barrett, Colin J. Suckling, Fraser J. Scott

**Affiliations:** WestCHEM Department of Pure and Applied Chemistry, University of Strathclyde Glasgow UK fraser.j.scott@strath.ac.uk; Wellcome Centre for Integrative Parasitology, Institute of Infection, Immunity and Inflammation and Glasgow Polyomics, College of Medical, Veterinary and Life Sciences, University of Glasgow Glasgow UK; Parasite Chemotherapy Unit, Department of Medical Parasitology and Infection Biology, Swiss Tropical and Public Health Institute Basel Switzerland; University of Basel Basel Switzerland; Strathclyde Institute of Pharmacy and Biomedical Science, University of Strathclyde Glasgow UK

## Abstract

This paper describes the design and synthesis of Strathclyde minor groove binders (S-MGBs) that have been truncated by the removal of a pyrrole ring in order to mimic the structure of the natural product, disgocidine. S-MGBs have been found to be active against many different organisms, however, selective antiparasitic activity is required. A panel of seven truncated S-MGBs was prepared and the activities examined against a number of clinically relevant organisms including several bacteria and parasites. The effect of the truncation strategy on S-MGB aggregation in aqueous environment was also investigated using 1H inspection and DOSY experiments. A lead compound, a truncated S-MGB, which possesses significant activity only against trypanosomes and *Leishmania* has been identified for further study and was also found to be less affected by aggregation compared to its full-length analogue.

## Abstract 2

This paper describes the design and synthesis of Strathclyde minor groove binders (S-MGBs) that have been truncated by the removal of a pyrrole ring in order to mimic the structure of the natural product, disgocidine.

## Graphical abstract

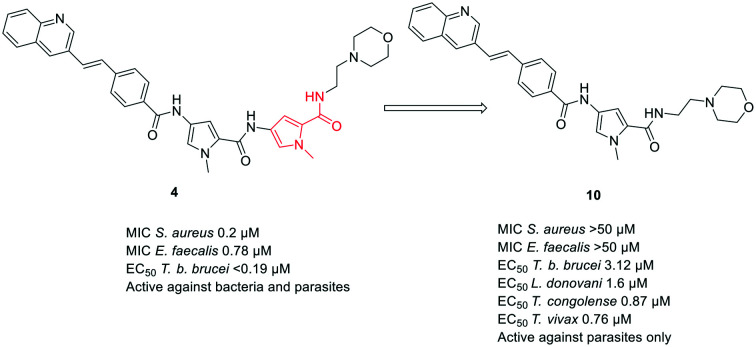

## 1. Introduction

Minor groove binders are a class of compound that interact with the minor groove of DNA. They have been shown to have a broad range of activities against many biological targets, including bacteria, fungi, parasites and cancer cells.^1,2^ The design paradigm at the University of Strathclyde has been to synthesise compounds based loosely upon the structure of the natural product, distamycin, **1**, with antibacterial activity being at the forefront of compound selection.^3^ This has resulted in a lead set of compounds in which the most notable changes from distamycin are the N-terminal formyl group being replaced by an aromatic ring, one of the amide links being replaced by the isosteric alkene, and the replacement of the amidine with a morpholine, as represented by **2–4**.^4^ These changes have been based on the desire to minimise toxicity, modulate lipophilicity and maximise antibacterial activity. Compounds **2–4** are all significantly active against Gram-positive bacteria and are in clinical development against both MRSA and *C. difficile*, with compound **4** having completed phase IIa against the latter.^5^ Although the full mechanism of antibacterial activity of these compounds has not yet been established, there is strong evidence that it is novel, involving the inhibition of several DNA-centric events.^6,7^

These compounds were selected for development because of their potent antibacterial properties; however, they also possess activity against a number of other species, including various parasites.^7–11^ This study is concerned with exploiting the anti-parasitic activity present within this panel of compounds. Activity against multiple organisms presents a potential issue as our S-MGBs in clinical development exert their antibacterial effects through a novel mechanism of action and we may not want to compromise this potential clinical impact by developing compounds with broad-spectrum activity and a common mechanism of action in other therapeutic areas. Consequently, our aim was to identify a different subclass of S-MGB that did not have antibacterial activity so as to minimise the potential for developing antibacterial resistance if other S-MGBs were eventually used as antiparasitics. We have found that whilst DNA binding is necessary for activity, it is not sufficient, and overall physicochemical properties of S-MGBs can affect internalization within pathogen cells to a different extent to afford different pathogen activity profiles.^6,7^ This study thus investigates modifications of the structures of our lead compound set, without compromising DNA binding, to remove the antibacterial activity whilst retaining activity against parasites, namely *Trypanosoma brucei*, *T. congolense*, *T. vivax* and *L. donovani*.

**Fig. 1:**
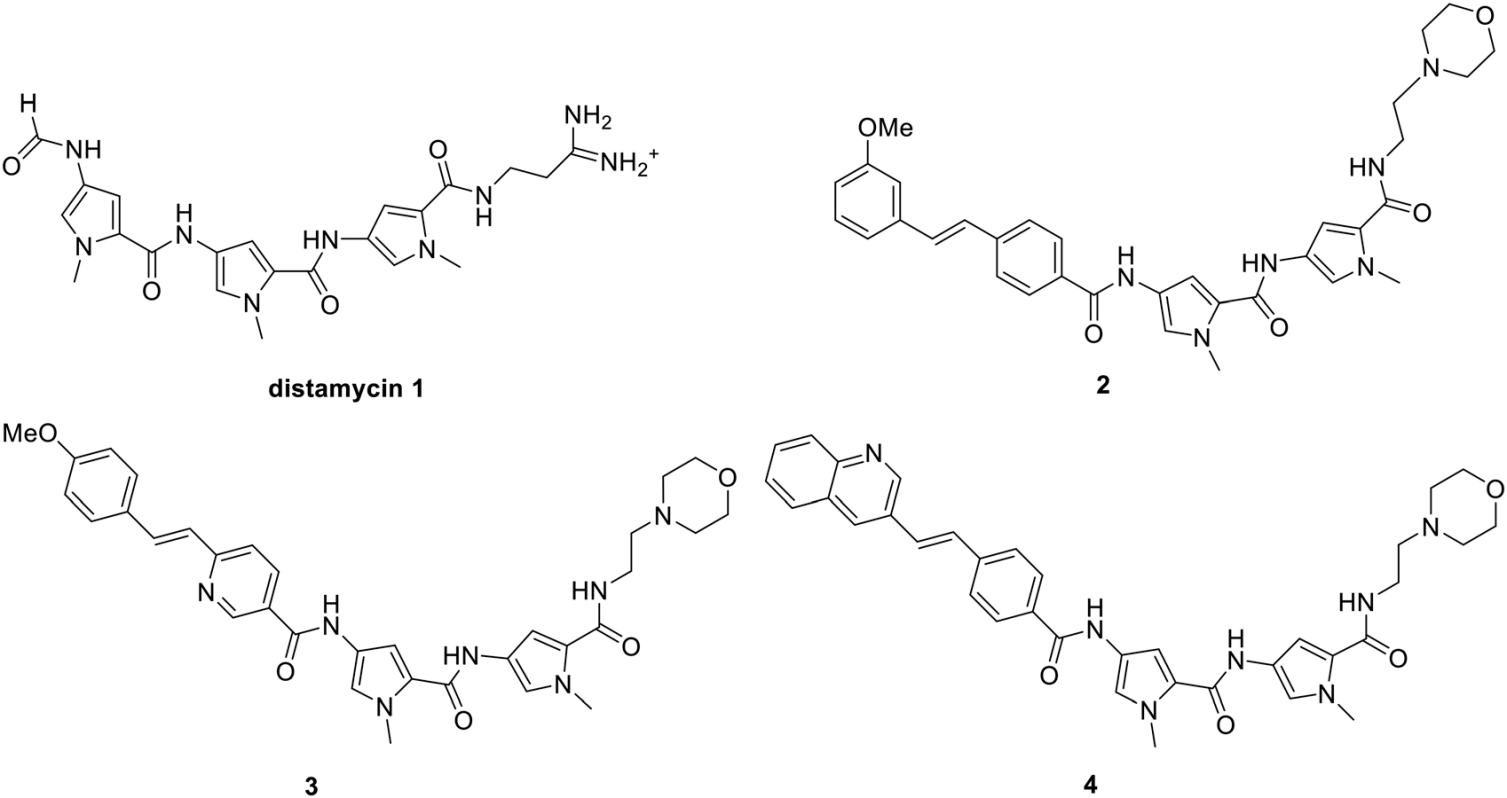
Structures of distamycin and lead antibacterial S-MGBs.

Furthermore, the lead compounds in clinical development as antibacterial agents require careful formulation due to gelation at concentrations of clinical relevance. Strong anti-parallel dimerization in dilute aqueous solution has been demonstrated for alkene-containing MGBs such as **2** and it is this, and further aggregation, that we believe leads to the undesired gelation.^12^ Thus, an added consideration in this study is to make structural changes in order to minimise the self-assembly and consequently mitigating potential solubility issues (Fig. 1).

**Fig. 2:**
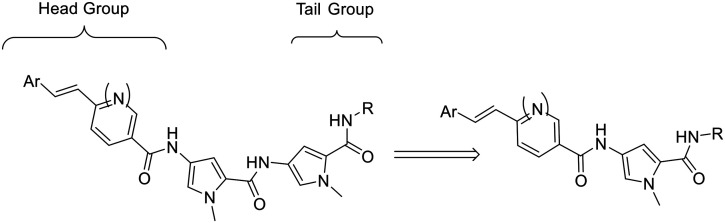
Illustration of the truncation strategy.

The S-MGBs in our lead compound set are conceptually composed of a bis-aromatic alkene head group, two pyrrole rings and a basic nitrogen containing tail group, all linearly connected *via* amide links. Recently, a new natural product, disgocidine, was identified from *Streptomyces netropsis* DSM 40846, which also produces distamycin, the compound on which our S-MGBs are based.^13^ The structure of disgocidine matches distamycin exactly, except that it contains one fewer pyrrole in its structure. Taking inspiration from disgocidine, for this study, we opted to truncate the length of our S-MGBs through removing one of the pyrrole rings (Fig. 2).

This truncation strategy would reduce the likelihood of molecular aggregation by reducing the strength of intermolecular dimerization due to fewer intermolecular forces between dimers. Moreover, these smaller S-MGBs would be more comparable in design, and overall physicochemical properties, to the diamidine class of MGBs, a class that are well-known for their potent antiparasitic activities, such as furamidine, pentamidine, DB75 or DB289.^1,2,7^

## 2. Chemistry

**Fig. 3:**
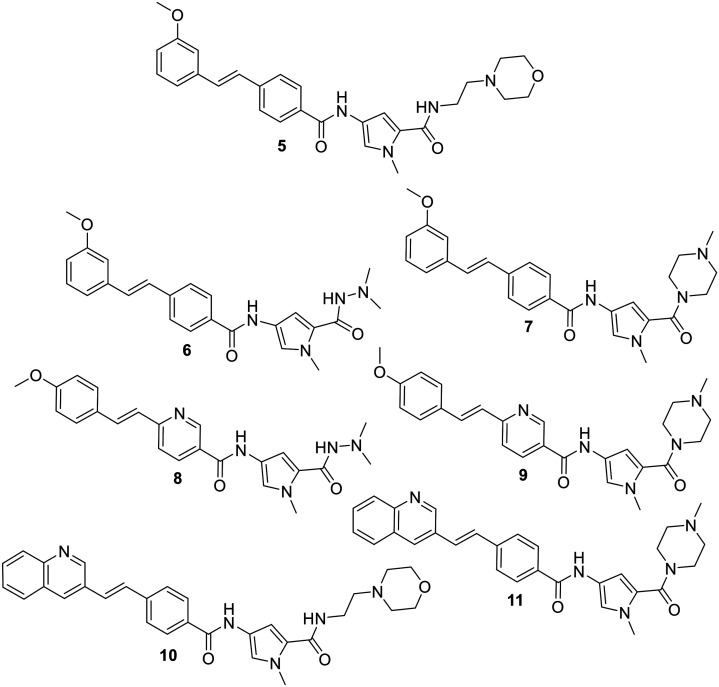
Truncated S-MGBs investigated in this study.

In order to evaluate the truncation strategy, a set of seven compounds was designed in which the structures explored several different head groups and tail groups typical of our S-MGBs. In addition to the morpholine containing tail group that is common to compounds **2–4**, two other tail groups, an *N*-methylpiperazine and *N*,*N*-dimethylhydrazine, were included, to increase the chemical space explored (Fig. 3).

**Scheme 1:**
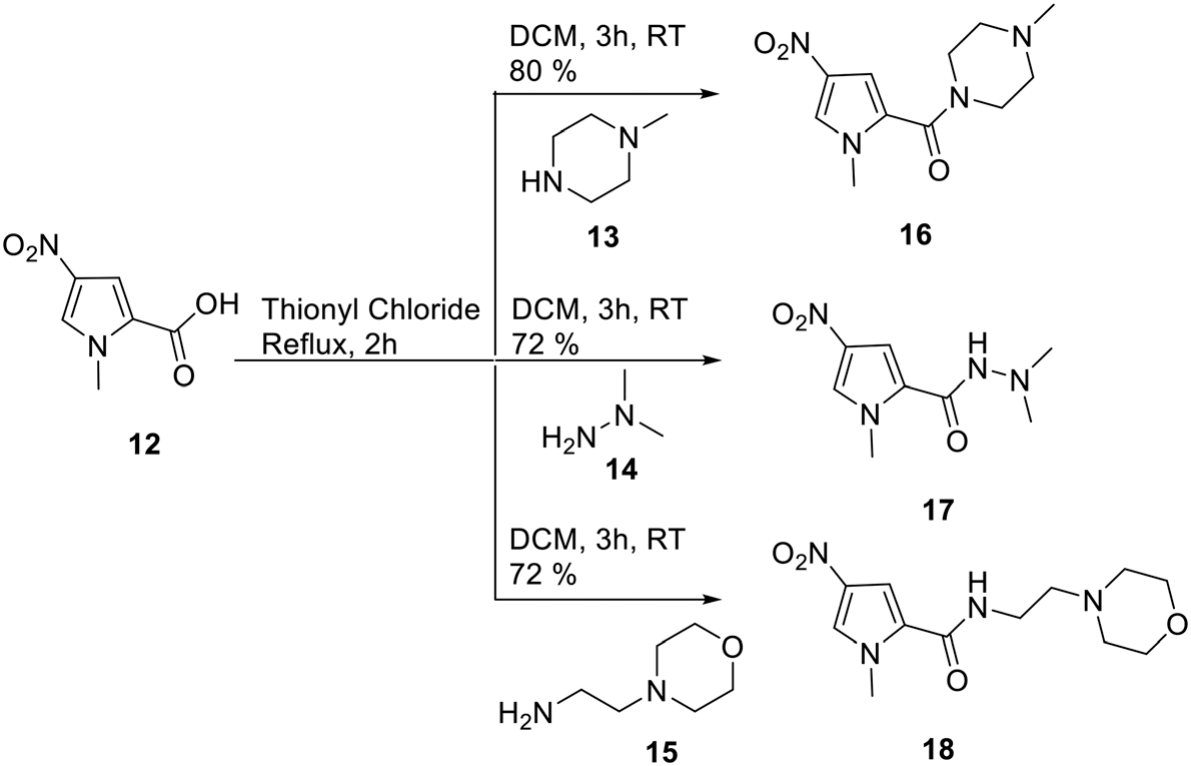
Synthesis of tail group monomers.

**Scheme 2:**
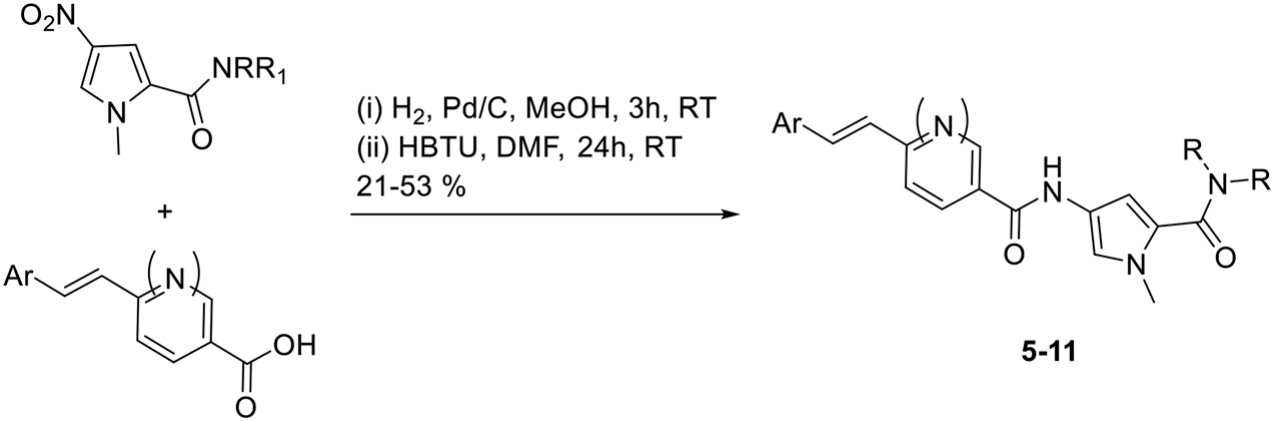
Final coupling to form S-MGBs.

The synthesis of these compounds followed the standard S-MGB synthesis that has been routinely described. Briefly, the tail group monomer was constructed by forming the acid chloride of the nitropyrrole **12** and reacting this with 2 eq. of either *N*-methylpiperazine (**13**), *N*,*N*-dimethylhydrazine (**14**), or aminoethylmorpholine (**15**), to form **16**, **17** and **18**, respectively (Scheme 1). An equivalent of amine was used to quench the HCl formed during the reaction. The nitro group of the tail group monomer, **16**, **17**, or **18**, was reduced to the corresponding amine (Pd/C) and subsequently coupled with the previously reported head group carboxylic acids using HBTU.^14^ This yielded all the required truncated MGBs, **5–11**, which were purified by HPLC (Scheme 2).

## 3. Biological activity

The parent compounds have previously been shown to have significant anti-Gram-positive bacterial activity in addition to a range of anti-trypanosomal activities.^3,7^ The newly synthesized compounds were initially evaluated for their antibacterial activity against two strains of Gram-positive bacteria, *Staphylococcus aureus*, ATCC 43300 and *Enterococcus faecalis*, ATCC 51299, and the parasite *Trypanosoma brucei brucei*, Lister 427. Vancomycin and diminazene were used as controls for bacterial and trypanosomal activity, respectively (Table 1).

**Table 1:** *In vitro* activity of S-MGBs and reference compounds against MRSA and *T. b. brucei*

Compound	*S. aureus* MIC (μM)	*E. faecalis* MIC (μM)	*T. b. brucei* EC_50_ (μM)
**2**	6.25	25	0.78
**3**	1.56	3.12	1.56
**4**	0.2	0.78	<0.19
**5**	>50	>50	>50
**6**	>50	>50	>50
**7**	>50	>50	>50
**8**	>50	>50	>50
**9**	>50	>50	>50
**10**	>50	>50	3.12
**11**	>50	>50	6.25
Vancomycin	0.39	6.25	—
Diminazene	—	—	0.5

The most obvious feature of the activities presented in Table 1 is the complete loss of antibacterial activity for all of the truncated MGBs; however, the truncation strategy has not removed all activity. Both compound **10** and compound **11** have retained modest activity against *T. b. brucei*. This discovery prompted a more detailed investigation of the antiparasitic activities of these compounds including an evaluation of activity against two further species of animal trypanosomes, *T. congolense* IL3000 and *T. vivax* STIB719 and another kinetoplastid parasite, *Leishmania donovani*, responsible for visceral leishmaniasis in humans. As a measure of general cytotoxicity, activity against the rat myoblast cell line, L6 was assessed. Amphotericin B and diminazene were used respectively as controls for anti-leishmanial and anti-trypanosomal activity (Table 2).

**Table 2:** *In vitro* activity of selected S-MGBs and reference compounds against *L. donovani*, *T. congolense*, *T. vivax* and L6 rat myoblast cell line (mean ± SEM, *n* ≥ 3). The EC_50_ value was calculated from 3 separate experiments

Compound	EC_50_ (μM)			
	*L. donovani*	*T. congolense*	*T. vivax*	L6
**10**	1.6 ± 0.90	0.87 ± 0.21	0.76 ± 0.13	6.7 ± 1.1
**11**	NA	6.6 ± 0.8	6.9 ± 1.2	41 ± 3.1
Amphotericin B	0.10 ± 0.00	—	—	—
Diminazene	—	0.13 ± 0.02	0.17 ± 0.03	—

Compound **11** had an EC_50_ of 6.6 μM and 6.9 μM against *T. congolense* and *T. vivax*, respectively, which is significant given its truncated structure. The most interesting compound of the set is compound **10**, which displays approximately an order of magnitude superior activity against *T. congolense* and *T. vivax* and excellent activity against *L. donovani*. The activity of compound **10** against *L. donovani* with an EC_50_ of 1.6 is significant. Using the activity against the L6 cell line as a measure of mammalian cell toxicity of compound **10** gives selectivity indices of 4.2, 7.7 and 8.2 against *L. donovani*, *T. congolense* and *T. vivax*, respectively. This level of selectivity is a positive outcome; however, this compound is not optimised and understanding from our ongoing S-MGB development programme could be used to reduce the potential toxicity as has recently been demonstrated.^7^

Considering the chemical structures, it is clear that there exists an important role for the quinoline head group in terms of the biological activities: both compounds that retain activity upon truncation contain this head group. Previous studies have demonstrated that the quinoline head group has a profound effect on enhancing the DNA binding strength of our full-length S-MGBs.^3^ Whilst the truncation strategy will have most likely reduced the DNA binding strength, due to the smaller molecules having less potential to make favourable hydrogen bonding or hydrophobic contacts with the minor groove, those with the quinoline head group, having a higher binding strength initially, appear to have retained enough DNA binding to afford activity. To investigate this hypothesis, a DNA thermal melting study was carried out.

The new truncated compounds. **5–11**, along with their parent compounds, **2–4**, were evaluated for their relative DNA binding affinities to a DNA oligomer with base sequence 5′-GCAAATTTCG-3′ and its complement 5′-CGTTTAAAGC-3′ by measuring their effects on its melting temperature. The results are shown in Table 3 below. It is clear from these data that the truncation of S-MGBs has eliminated DNA binding, except in those compounds that contain the quinoline head group. The melting temperature increase difference between compounds **10** and **11** also indicates the importance of the tail group in these compounds: the morpholine tail compound, **10**, has a 5-fold greater melting temperature increase compared to the *N*-methylpiperazine compound, **11**. This may be counter intuitive as **10** possesses the more flexible tail group, but perhaps this allows for better interaction between its basic nitrogen and the phosphate backbone.

**Table 3:** DNA melting temperature increase against the oligomer 5′-GCAAATTTCG′3′/5′-CGTTTAAAGC-3′

S-MGB	**2**	**3**	**4**	**5**	**6**	**7**	**8**	**9**	**10**	**11**
Δ*T*_m_/°C	10	12	20	0	0	0	0	0	10	2

The development of S-MGBs as selective anti-parasitic agents, in particular as trypanocides, could be hindered by cross-resistance to the diamidine class of compounds, which have been routinely used as antiparasitics for decades, as these are also thought to function through binding to the minor groove of DNA.^15–17^ In our previous study of S-MGBs as potential agents to treat animal African trypanosomiasis, we demonstrated that full length S-MGBs did not demonstrate cross-resistance.^7^ To assess this risk in these novel truncated S-MGBS, we tested compounds **10** and **11** on two diminazene-resistant *T. congolense* lines selected *in vitro*. The same S-MGBs were also tested on two *T. b. brucei* lines lacking the amino–purine transporter TbAT1/P2,^18^ which carries diamidines, and the aquaglyceroporin HAPT1/AQP2,^19^ known to be associated with pentamidine uptake in *T. brucei*, and whose loss is linked to resistance and cross-resistance onset in these organisms (Table 4).

*In vitro* trypanocidal activity of **10** and **11** against two diminazene-resistant *T. congolense* lines (DimR and EMS MUT DimR) as compared to wild type (WT) and *T. b. brucei* lines lacking TbAT1/P2 (*tbat1*^−/−^) and TbAT1/P2 plus HAPT1/AQP2 (B48), as compared to wild type (WT) (mean ± SEM, *n* ≥ 3). RF, resistance factor

**Table:** 

	*T. congolense* EC_50_ (μM)				
Compound	WT	DimR	RF	EMS MUT DimR	RF
**10**	3.4 ± 0.4	3.1 ± 0.2	0.9	3.2 ± 0.3	0.9
**11**	4.3 ± 0.7	4.0 ± 0.9	0.9	4.5 ± 0.6	1.0
Diminazene	0.2 ± 0.01	2.1 ± 0.1	9.5	2.3 ± 0.1	10.9

**Table:** 

	*T. b brucei* EC_50_ (μM)				
	WT	*tbat1* ^−/−^	RF	B48	RF
**10**	1.2 ± 0.03	0.55 ± 0.04	0.5	0.6 ± 0.1	0.5
**11**	2.7 ± 0.6	2.1 ± 0.6	0.8	2.7 ± 0.6	1.0
Diminazene	0.03 ± 0.01	0.18 ± 0.02	5.9	0.08 ± 0.01	2.6

The results showed no loss of activity against the diminazene-resistant *T. congolense* lines for any of the compounds indicating a lack of cross-resistance. Furthermore, no changes to EC_50_ values for the S-MGBs against the *T. b. brucei* mutants were observed in the absence of TbAT1/P2 and HAPT1/AQP2 transporters, confirming that the S-MGBs are not substrates for these carriers. Taken together, these results show that the S-MGBs are unlikely to suffer cross-resistance in parasites resistant to diamidines, despite both classes of compound binding the minor groove of DNA. This could be explained by the two classes of compounds entering the cells *via* different routes of uptake.

## 4. Aggregation studies

**Fig. 4:**
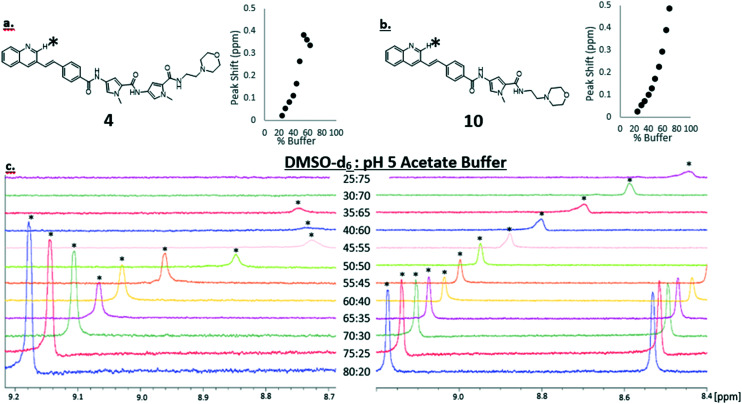
a) Structure of compound **4** with a graph of peak shift, relative to the ppm in 80% DMSO-*d*_6_/20% 0.1 M pH 5 sodium acetate buffer, of the quinoline proton marked by an asterisk. b) Structure of compound **10** with a graph of peak shift, relative to the ppm in 80% DMSO-*d*_6_/0.1 M 20% pH 5 sodium acetate buffer, of the quinoline proton marked by an asterisk. c) ^1^H NMR of a quinoline proton of compound **4** (left) and compound **10** (right). The quinoline proton marked by an asterisk is shown to shift between 9.2–8.4 ppm as the composition of the solvent (DMSO-*d*_6_ and 0.1 M pH 5 acetate buffer). The concentration of DMSO-*d*_6_ in each sample was diluted by 5% from 80% DMSO-*d*_6_, with 0.1 M pH 5 sodium acetate buffer. A fixed concentration of 1.5 mM of S-MGB **4** or **10** was maintained for each experiment.

We know that the propensity for S-MGBs to self-associate can lead to poor solubility profiles, which is not desirable for any potential drug. Thus, we sought to use NMR techniques to obtain an indication of likely solubility issues of a truncated S-MGB through measuring the degree of self-association. Compound **10** was selected for this investigation due to its more favourable activity profile and its full-length analogue, compound **4**, was selected as a reference. As has been observed in previous S-MGBs, both compounds displayed significant line broadening in an aqueous buffered system (0.1 M pH 5 sodium acetate) compared to DMSO-*d*_6_ – this is indicative of a system involving aggregation (S2†). For **4**, this was to the extent that no signals could be observed. We first sought to compare this phenomenon between each compound by systematically acquiring ^1^H-NMR spectra in a solvent system with different proportions of DMSO-*d*_6_ and pH 5 sodium acetate buffer, using a fixed concentration of 1.5 mM of S-MGB. Fig. 4 shows an extract of this data focusing on a quinoline proton in each compound, but the full data sets can be found in the ESI† (S1–S5 and S7–S11).

**Fig. 5:**
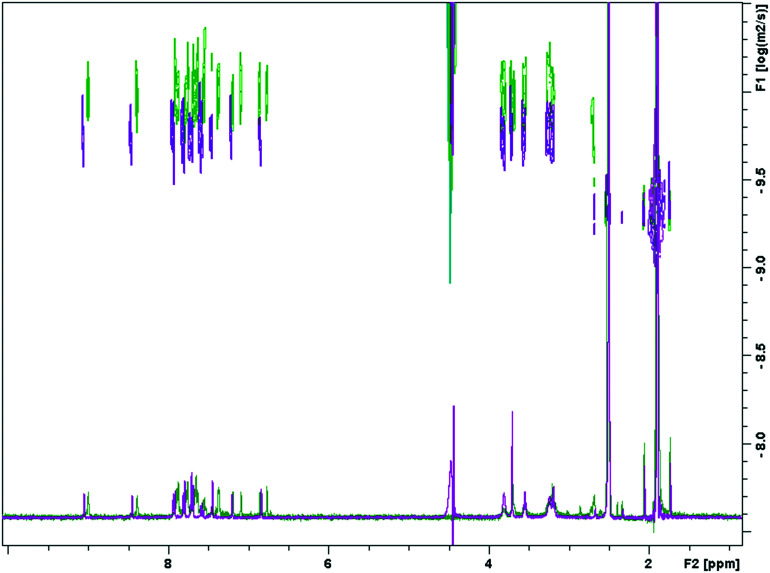
DOSYs of compound **4** (green) and compound **10** (purple).

For both compounds, there can be seen a change in ppm of the chemical shift as the proportion of aqueous buffer is increased. In general, this is an upfield shift, likely caused by a shielding effect from the π–π stacking of the planar molecules as the aggregate grows. However, the spectrum does exhibit more complex behaviour which could allude to a change in aggregation behaviour at a critical solvent composition. The quinoline proton on both **4** and **10** both move upfield as the proportion of aqueous buffer increases. However, upon reaching 55% buffer composition, the quinoline proton of **4**, (Fig. 4, panel a), begins to shift downfield. This more complex behavior was not observed in the truncated MGB, **10**, and again could be indicative of an increased aggregation of **4** relative to **10**. Significantly, for the full S-MGB, **4**, the quinoline signals can no longer be observed at 70% v/v buffer; however, for the truncated S-MGB, **10**, the signal can still be observed in the highest percentage of buffer used in the experiment (S8†). This difference in behavior is in line with the truncated S-MGB forming smaller aggregates in aqueous environments. To confirm this, we also carried out a comparative DOSY experiment in which we selected a solvent 1q system of 50% v/v pH 5 acetate buffer in DMSO-*d*_6_ for a concentration of 1.5 mM of each S-MGB (Fig. 5). In this experiment, compound **10** had a diffusion coefficient of 1.753 × 10^−10^ ± 0.048 × 10^−10^, whereas the diffusion coefficient of compound **4** was calculated to be 1.0678 × 10^−10^ ± 0.0612 × 10^−10^. This again confirms that the truncation strategy has had the desired effect of reducing the size of S-MGB aggregates.

## 5. Conclusion

The purpose of this study was to determine if a truncation strategy, whereby a pyrrole is removed from the length of an S-MGB, can reduce antibacterial activity whilst maintaining activity against parasites. Truncation has been shown to remove antibacterial activity, even from compounds that are highly effective antibacterial therapeutics, and this is likely due to a combination of a change in physicochemical properties affecting intracellular accumulation and a reduced affinity for DNA. The reduction in antibacterial activity through the truncation strategy has not entirely compromised the activity against the other organisms under study; moreover, compound **10** has significant activity against *L. donovani* and developable activity against *T. congolense* and *T. vivax*. For these compounds, some DNA binding capability has been retained, sufficient for activity, and it is likely that the resultant physicochemical properties of the truncated molecules have not suppressed their ability to enter parasite cells, only bacterial cells. Furthermore, confirmation was obtained that no cross-resistance is expected with the diamidine class of compounds as previous data already indicated.^7^ We have also confirmed that the truncation strategy has the added beneficial effect of reducing the extent of S-MGB aggregation in aqueous environments, likely reducing the potential of problematic solubility profiles. Compound **10** thus represents a structure that can be used as a lead for further optimisation in the design of selective antiparasitic S-MGBs, *i.e.*, those that do not possess antibacterial activity.

## Experimental

### Antibacterial assay

The minimum inhibitory concentration (MIC) against *S. aureus* ATCC 43300 and *E. faecalis* ATCC 51299 was measured by making two-fold serial dilution of the samples into 96-well non-binding surface plate (Corning #3640). Bacteria were cultured in cation-adjusted Mueller Hinton broth (CAMHB) overnight at 37 °C and diluted 40-fold and incubated for a further 1.5–3 h at 37 °C. The resultant mid-log phase cultures were diluted and added to each well of the compound containing plates, giving a cell density of 5 × 10^5^ CFU mL^−1^, measured by absorbance at 600 nm (OD600), and a final compound concentration range of 50–0.0195 μM. All the plates were covered and incubated at 37 °C for 18 h without shaking. Inhibition of bacterial growth was determined by OD600, using a Tecan Infinite M Nano plate reader. The percentage of growth inhibition was calculated for each well, using the negative control (medium only) and positive control (bacteria without inhibitors) on the same plate. The MIC was determined as the lowest concentration at which the growth was fully inhibited, defined by an inhibition 80%. Each MIC determination was carried out in triplicate, on separate days.

### Anti-*Trypanosoma* assay

Bloodstream form *T. b. brucei* (Lister 427) and derived lines *tbat1*^−/−^ and B48 were cultured in HMI-11 medium (Gibco) supplemented with 10% heat inactivated FBS (Gibco), at 37 °C in a humidified 5% CO_2_ environment. Bloodstream form *T. congolense* (strain IL3000) and derived diminazene resistant lines were cultured at 34 °C in a humidified, 5% CO_2_ environment in MEM medium (Sigma-Aldrich), supplemented with 25 mM HEPES, 26 mM NaHCO_3_, 5.6 mM d-glucose, 1 mM sodium pyruvate, 40 μM adenosine, 100 μM hypoxanthine, 16.5 μM thymidine and 25 μM bathocuproinedisulfonic acid disodium salt, β-mercaptoethanol (0.0014% v/v), 1.6 mM glutamine, 100 units per ml penicillin, 0.1 mg ml^−1^ streptomycin, 20% goat serum (Gibco) and 5% Serum Plus (SAFC Biosciences). Selection of the *T. congolense* diminazene resistant lines was carried out as previously described.^7^*T. Vivax* (STIB 719/ILRAD 560 strain) culture was also carried out as previously described.^20^

EC_50_ values against *T. b. brucei* and *T. congolense* were determined by the *in vitro* Alamar Blue assay. *T. b. brucei* parasites (2 × 10^4^ cells per mL), or *T. congolense* (2.5 × 10^5^ cells per mL) were seeded into serial dilutions of the test compounds to a final volume of 200 μl and incubated for 48 h after which, 20 μL of 0.49 mM resazurin dye (Sigma Aldrich) was added and cells were incubated for a further 24 h. The reduction of resazurin was measured using a fluorimeter (FLUOstar Optima, BMG Labtech) at 544 nm excitation and 590 nm emission wavelengths. EC_50_ values were determined using Prism 5 software (GraphPad). All experiments were carried out on at least three independent occasions. EC_50_ values against *T. vivax* were determined using the *ex vivo* 3H-hypoxanthine incorporation assay.^7^

### Anti-*Leishmania* assay

Compounds were screened *in vitro* for their antileishmanial activity against the intracellular amastigote stage.^21^ Bone marrow derived macrophages (0.5 × 10^5^ cells per well) were infected using a 20 : 1 host cell : parasite ratio with luciferase-expressing *L. donovani* LV82 luc1 (MHOM/ET/67:LV82). The medium was changed at 24 post-infection to remove free parasites and infected cells were treated with medium alone (controls, *n* = 6) or doubling dilutions of DMSO (*n* = 3, starting at 2% or 2.5% v/v) or MGB compounds (*n* = 3 or 6, starting at 25 or 20 μg mL^−1^, prepared using a 1 mg mL^−1^ DMSO stock solution). Amphotericin B solution was used as a positive control and was used at a starting concentration of 1 μM. The medium was removed from each sample at 72 hours post-infection and replaced with 150 μl luciferin solution (150 μg mL^−1^ luciferin in serum free medium). The amount of bioluminescent signal emitted/well was determined and used to calculate the mean suppression in the bioluminescent signal for each test sample compared to the mean control values. The EC_50_ values were calculated by probit analysis.^22^ The mean EC_50_ values were calculated from 3 separate experiments.

### L6 toxicity assay

To assess the cytotoxicity of tested compounds, the Alamar Blue assay was followed using the L6 (rat skeletal myoblast) cell line.^7^ All experiments were performed in three independent assay runs for each compound.

### DNA thermal melting study

DNA oligomers and their complements were melted at a rate of 0.5 °C min^−1^ in 10 mM PBS buffer solution (pH 7.4) with 50 mM NaCl on a Cary 300 BIO UV-visible spectrophotometer fitted with a Peltier temperature controller. Programs were set and data was processed using Cary WinUV software. The duplex oligomer was made to a concentration of 6 × 10^−6^ M, heating to 90 °C and allowed to cool to room temperature unaided. This was mixed with sufficient S-MGB to give the appropriate ratio 2 S-MGB : 1 DNA helix. For the reference melting temperature, no MGB was added. Samples were heated from 10 °C to 90 °C and cooled from 80 °C to 10 °C with the spectra being recorded at 260 nm during both of these cycles. The melting temperatures (*T*_m_) of the duplex oligomers were determined by fitting a sigmoidal function using a Boltzmann distribution in OriginPro. This process was repeated a total of 3 times to ensure repeatability of the experiment which showed an average error of ±0.5 °C.

### Chemical analysis

#### General experimental methods

^1^H and ^13^C NMR spectra were measured on a Bruker DPX-500 MHz spectrometer with chemical shifts given in ppm (*d* values), relative to proton and carbon traces in solvent. Coupling constants are reported in Hz. The data were presented as follows: chemical shift, multiplicity (s = singlet, d = doublet, t = triplet, q = quartet, m = multiplet, br = broad, app = apparent), coupling constant (s) in Hertz (Hz), and integration. Chemical shifts (*δ*) were recorded relative to residual DMSO-*d*_6_ (*δ* = 2.50 in ^1^H NMR and *δ* = 35.2 in ^13^C NMR). IR spectra were recorded on a Perkin Elmer, 1 FT-IR spectrometer. Mass spectra were obtained on a Jeol JMS AX505. Anhydrous solvents were obtained from a Puresolv purification system, from Innovative Technologies, or purchased as such from Aldrich. Melting points were recorded on a Reichert hot-stage microscope, and are uncorrected. Chromatography was carried out using 200–400 mesh silica gels, or using reverse-phase HPLC on a water system using a C18 Luna column and gradient given in individual entries below. The purity of all new S-MGBs was greater than 96%, which was confirmed by HPLC.

#### General tail group monomer synthesis

1-Methyl-4-nitro-1*H*-pyrrole-2-carboxylic acid **12** (100 mg, 0.64 mmol) was added to thionyl chloride (5 mL) and refluxed for 2 h. The excess thionyl chloride was then removed under reduced pressure and the residue dissolved in DCM (5 mL, dry); to this was added the amine, **13–15** (2.5 equiv.) and then left to stir for 3 h. The DCM was washed with water (2 × 5 mL) and the volume of the organic layer was reduced under reduced pressure and the resulting product was obtained by filtration.

#### 1-methyl-4-[(1-methyl-4-nitro-1*H*-pyrrol-2-yl)carbonyl]piperazine **16**

Light brown powder. Yield 80%, mp > 230 °C; IR (KBr, cm^−1^): 3375, 3176, 3025, 2496, 2427, 1625, 1588, 1547, 1508, 1461, 1427, 1299; ^1^H NMR (DMSO-*d*_6_, 500 MHz) *δ*: 8.13 (1H, d, *J* = 2.0), 6.90 (1H, d, *J* = 2.0), 3.72 (3H, s), 3.60 (4H, m), 2.38 (4H, m), 2.23 (3H, s). ^13^C NMR (DMSO-*d*_6_, 125 MHz) *δ*: 159.8, 134.0, 126.6, 126.3, 106.3, 54.3, 45.3, 35.9, 30.63. HRMS (FAB) calcd. for C_11_H_17_N_4_O_3_ [M + H]^+^, 253.1295; found, 253.1281.

#### *N*′,*N*′,1-Trimethyl-4-nitro-1*H*-pyrrole-2-carbohydrazide **17**

Light brown powder. Yield 72%, mp > 230 °C; IR (KBr, cm^−1^): 3180, 3139, 2835, 2792, 1677, 1651, 1560, 1532, 1494, 1310, 1075; ^1^H NMR (DMSO-d_6_, 500 MHz) *δ*: 10.32 (1H, bs), 8.19 (1H, d, *J* = 2.0), 7.48 (1H, d, *J* = 2.0), 3.90 (3H, s), 2.74 (6H, s); ^13^C NMR (DMSO-*d*_6_, 125 MHz) *δ*: 157.7, 133.8, 128.6, 123.9, 108.5, 46.4, 37.3. HRMS (FAB) calcd. for C_8_H_13_N_4_O_3_ [M + H]^+^, 213.0982; found, 213.0988.

#### 1-Methyl-*N*-[2-(4-morpholinyl)ethyl]-4-nitro-1*H*-pyrrole-2-carboxamide **18**

Light brown powder. Yield 72%, mp 140–141 °C; IR (KBr, cm^−1^): 3326, 3118, 2928, 2816, 1634, 1551, 1533, 1146, 1114; ^1^H NMR (DMSO-d_6_, 500 MHz) *δ*: 7.56 (1H, d, *J* = 1.9), 7.06 (1H, d, *J* = 1.9), 6.58 (1H, bs), 4.00 (3H, s), 3.76 (4H, t, *J* = 4.6), 3.49 (2H, m), 2.59 (2H, t, *J* = 6.0), 2.51 (4H, t, *J* = 4.6); ^13^C NMR (DMSO-*d*_6_, 125 MHz) *δ*: 159.7, 133.7, 127.8, 126.4, 107.2, 66.15, 57.3, 53.2, 37.3, 35.9. HRMS (FAB) calcd. for C_12_H_19_O_4_N_4_ [M + H]^+^, 283.1406; found, 283.1411.

### General truncated S-MGB synthesis

The tail group monomer **16–18** (0.14 mmol) was dissolved in MeOH (5 mL) and Pd/C-10% was added under nitrogen (15 mg). This was subjected to hydrogenation for 3 h. After this the solution was filtered through kieselguhr, reduced under vacuum, and the resultant residue dissolved in DMF (1 mL, dry). The appropriate carboxylic acid (0.14 mmol) was dissolved in anhydrous DMF (1 mL), to which was added HBTU (0.28 mmol) and triethylamine (0.28 mmol) and left to stir for 30 min. The DMF solution containing the amine was then added to the that containing the active ester and then left to stir for 16 h. The reaction mixture was subjected to HPLC purification to obtain the desired product.

#### 4-[2-({[4-({4-[(*E*)-2-(3-Methoxyphenyl)ethenyl]benzoyl}amino)-1-methyl-1*H*-pyrrol-2-yl]carbonyl}amino)ethyl]morpholin-4-ium trifluoroacetate **5**

Off-white fluffy powder; yield 20%; no distinct melting point; IR (KBr, cm^−1^): 3424, 3026, 2928, 2857, 2604, 2483, 1675, 1650, 1606, 1577, 1559, 1536, 1464, 1437, 1402, 1274, 1202, 1131, 1045, 1015; ^1^H NMR (DMSO-d_6_, 500 MHz) *δ*: 10.32 (1H, s), 9.72 (1H, bs), 8.27 (1H, m), 7.96 (2H, d, *J* = 8.4), 7.74 (2H, d, *J* = 8.4), 7.31–7.38 (4H, m), 7.21–7.23 (2H, m), 7.03 (1H, d, *J* = 1.6), 6.89 (1H, dd, *J* = 8.0, J = 2.0), 3.95–4.04 (2H, m), 3.86 (3H, s), 3.81 (3H, s), 3.63–3.67 (2H, m), 3.51–3.58 (4H, m), 3.26–3.29 (2H, m), 3.12–3.15 (2H, m); ^13^C NMR (DMSO-*d*_6_, 125 MHz) *δ*: 163.2, 161.8, 159.6, 158.1, 139.8, 138.1, 133.2, 130.2, 129.7, 127.8, 126.3, 122.3, 122.2, 119.3, 118.8, 113.9, 111.7, 111.6, 104.9, 63.2, 55.6, 55.0, 51.3, 36.1, 33.2. HRMS (FAB) calcd. for C_28_H_33_O_4_N_4_ [M + H]^+^, 489.2496; found, 489.2496.

HPLC procedure: flow rate: 6 mL min^−1^. Retention time: 4.3 min

**Table:** 

Time (min)	% water (with 0.1% TFA)	% MeCN (with 0.1% TFA)
Isocratic	50	50

#### 2-{[4-({4-[(*E*)-2-(3-Methoxyphenyl)ethenyl]benzoyl}amino)-1-methyl-1*H*-pyrrol-2-yl]carbonyl}-1,1-dimethylhydrazinium trifluoroacetate **6**

Off-white fluffy powder; yield 20%; no distinct melting point; IR (KBr, cm^−1^): 3271, 2963, 2835, 1666, 1638, 1580, 1439, 1399, 1174, 1127; ^1^H NMR (DMSO-d_6_, 500 MHz) *δ*: 10.32 (1H, s), 10.02 (1H, bs), 8.31 (1H, s), 7.96 (2H, d, *J* = 8.4), 7.74 (2H, d, *J* = 8.4), 7.31–7.38 (4H, m), 7.21–7.23 (2H, m), 7.03 (1H, bs), 6.89 (1H, dd, *J* = 8.0, *J* = 2.0), 3.85 (3H, s), 3.81 (3H, s), 2.80 (6H, s). HRMS (FAB) calcd. for C_24_H_27_O_3_N_4_ [M + H]^+^, 419.2078; found, 419.2076.

HPLC procedure: flow rate: 6 mL min^−1^. Retention time: 8.7 min

**Table:** 

Time (min)	% water (with 0.1% TFA)	% MeCN (with 0.1% TFA)
0	60	40
25	50	50
30	30	70
35	70	30
40	70	30

#### 1-{[4-({4-[(*E*)-2-(3-Methoxyphenyl)ethenyl]benzoyl}amino)-1-methyl-1*H*-pyrrol-2-yl]carbonyl}-4-methylpiperazin-4-ium trifluoroacetate **7**

Off-white fluffy powder; yield 15%; no distinct melting point; IR (KBr, cm^−1^): 3425, 3274, 3026, 2940, 2836, 2722, 2614, 2481, 1677, 1638, 1606, 1575, 1464, 1445, 1413, 1399, 1258, 1201, 1125, 1048, 1026, 1015, 975; ^1^H NMR (DMSO-d_6_, 500 MHz) *δ*: 10.29 (1H, s), 9.94 (1H, bs), 7.96 (2H, d, *J* = 8.4), 7.74 (2H, d, *J* = 8.4), 7.31–7.38 (4H, m), 7.21–7.23 (2H, m), 6.89 (1H, dd, *J* = 8.0, *J* = 2.0), 6.58 (1H, d, *J* = 1.6), 4.41–4.45 (2H, m), 3.81 (3H, s), 3.70 (3H, s), 3.46–3.49 (2H, m), 3.27–3.33 (2H, m), 3.07–3.10 (2H, m), 2.84 (3H, s). HRMS (FAB) calcd. for C_27_H_31_O_3_N_4_ [M + H]^+^, 459.2391; found, 459.2386.

HPLC procedure: flow rate: 6 mL min^−1^. Retention time: 10.5 min

**Table:** 

Time (min)	% water (with 0.1% TFA)	% MeCN (with 0.1% TFA)
0	66	34
25	50	50
30	30	70
35	70	30
40	70	30

#### 1-{({4-[({6-[(*E*)-2-(4-Methoxyphenyl)ethenyl]-3-pyridiniumyl}carbonyl)amino]-1-methyl-1*H*-pyrrol-2-yl}carbonyl)}-1,1-dimethylhydrazinium trifluoroacetate **8**

Orange fluffy powder; yield 35%; no distinct melting point; IR (KBr, cm^−1^): 3259, 3100, 3051, 2901, 1699, 1639, 1571, 1564, 1451, 1210, 1120; ^1^H NMR (DMSO-d_6_, 500 MHz) *δ*: 10.28 (1H, s), 9.66 (1H, bs), 9.01 (1H, d, *J* = 2.0), 8.23 (1H, dd, *J* = 8.0, *J* = 2.0), 7.62–7.77 (4H, m), 7.40 (1H, d, *J* = 1.6), 7.25 (1H, d, *J* = 16.0), 6.98 (2H, d, *J* = 8.0), 6.58 (1H, d, *J* = 1.6), 4.39–4.50 (2H, m), 3.82 (3H, s), 3.63 (3H, s), 3.47–3.50 (2H, m), 3.26–3.32 (2H, m), 3.01–3.11 (2H, m), 2.81 (3H, s). HRMS (FAB) calcd. for C_23_H_25_O_3_N_5_ [M + H]^+^, 420.2030; found, 430.2040.

HPLC procedure: flow rate: 6 mL min^−1^. Retention time: 10.1 min

**Table:** 

Time (min)	% water (with 0.1% TFA)	% MeCN (with 0.1% TFA)
0	70	30
25	50	50
30	30	70
35	70	30
40	70	30

#### 1-({4-[({6-[(*E*)-2-(4-Methoxyphenyl)ethenyl]-3-pyridiniumyl}carbonyl)amino]-1-methyl-1*H*-pyrrol-2-yl}carbonyl)-4-methylpiperazin-4-ium bis(trifluoroacetate) **9**

Orange fluffy powder; yield 15%; no distinct melting point; IR (KBr, cm^−1^): 3262, 3114, 3051, 2939, 1698, 1668, 1591, 1573, 1446, 1192, 1127; ^1^H NMR (DMSO-d_6_, 500 MHz) *δ*: 10.33 (1H, s), 9.78 (1H, bs), 9.04 (1H, d, *J* = 2.0), 8.24 (1H, dd, *J* = 8.0, *J* = 2.0), 7.63–7.76 (4H, m), 7.35 (1H, d, *J* = 1.6), 7.24 (1H, d, *J* = 16.0), 6.99 (2H, d, *J* = 8.0), 6.56 (1H, d, *J* = 1.6), 4.41–4.45 (2H, m), 3.79 (3H, s), 3.69 (3H, s), 3.46–3.49 (2H, m), 3.27–3.33 (2H, m), 3.07–3.10 (2H, m), 2.84 (3H, s). HRMS (FAB) calcd. for C_26_H_30_O_3_N_5_ [M + H]^+^, 460.2343; found, 460.2340.

HPLC procedure: flow rate: 6 mL min^−1^. Retention time: 10.4 min

**Table:** 

Time (min)	% water (with 0.1% TFA)	% MeCN (with 0.1% TFA)
0	70	30
25	50	50
30	30	70
35	70	30
40	70	30

#### 3-[(*E*)-2-(4-{[(1-Methyl-5-{[(2-morpholin-4-ium-4-ylethyl)amino]carbonyl}-1*H*-pyrrol-3-yl)amino]carbonyl}phenyl)ethenyl]quinolinium bis(trifluoroacetate) **10**

Yellow fluffy powder; yield 20%; no distinct melting point; IR (KBr, cm^−1^): 3414, 2956, 2925, 2851, 2186, 1677, 1651, 1632, 1603, 1533, 1438, 1403, 1389, 1337, 1282, 1204, 1133, 1016; ^1^H NMR (DMSO-*d*_6_, 500 MHz) *δ*: 10.37 (1H, s), 9.52 (1H, br), 8.41 (1H, d, *J* = 8.5), 8.27 (1H, t, *J* = 8.0), 7.86–8.02 (8H, m), 7.77 (1H, m), 7.56–7.66 (2H, m), 7.31 (1H, d, *J* = 1.7), 7.04 (1H, d, *J* = 1.7), 3.98–4.03 (2H, m), 3.85 (3H, s), 3.62–3.68 (2H, m), 3.51–3.59 (4H, m), 3.27 (2H, m), 3.12 (2H, m). HRMS (FAB) calcd. for C_30_H_32_O_3_N_5_ [M + H]^+^, 510.2500; found, 510.2499.

HPLC procedure: flow rate: 6 mL min^−1^. Retention time: 14.5 min

**Table:** 

Time (min)	% water (with 0.1% TFA)	% MeCN (with 0.1% TFA)
0	90	10
25	50	50
30	30	70
35	90	10
40	90	10

#### 3-((*E*)-2-{4-[({1-methyl-5-[(4-methylpiperazin-4-ium-1-yl)carbonyl]-1*H*-pyrrol-3-yl}amino)carbonyl]phenyl}ethenyl)quinolinium bis(trifluoroacetate) **11**

Yellow fluffy powder; yield 21%; no distinct melting point; IR (KBr, cm^−1^): 3286, 2870, 2632, 2494, 1679, 1655, 1649, 1565, 1459, 1267, 1181; ^1^H NMR (DMSO-*d*_6_, 500 MHz) *δ*: 10.34 (1H, s), 9.79 (1H, bs), 9.27 (1H, d, *J* = 2.0), 8.56 (1H, d, *J* = 2.0), 7.99–8.06 (4H, m), 7.75–7.84 (3H, m), 7.61–7.71 (3H, m), 7.39 (1H, d, *J* = 1.6), 6.59 (1H, d, *J* = 1.6), 4.43–4.47 (2H, m), 3.70 (3H, s), 3.48–3.51 (2H, m), 3.28–3.35 (2H, m), 3.08–3.13 (2H, m), 2.85 (3H, s). HRMS (FAB) calcd. for C_29_H_30_O_2_N_5_ [M + H]^+^, 480.2394; found, 480.2395.

HPLC procedure: flow rate: 6 mL min^−1^. Retention time: 14.4 min

**Table:** 

Time (min)	% water (with 0.1% TFA)	% MeCN (with 0.1% TFA)
0	70	30
25	50	50
30	30	70
35	70	30
40	70	30

#### NMR aggregation experiments

The NMR techniques used to investigate the self-association behaviours of S-MGB's **4** and **10** involved obtaining standard ^1^H-NMRs of each S-MGB compound in a set variety of solvent system compositions. The concentration of DMSO-*d*_6_ in each sample was diluted by 5% starting from 80% DMSO-*d*_6_, with pH 5 sodium acetate buffer. Thus, twelve spectra for both S-MGB **4** and **10** were obtained with the following solvent system compositions: (A) 80% DMSO-*d*_6_: 20% pH 5 sodium acetate buffer, (B) 75% DMSO-*d*_6_: 25% pH 5 sodium acetate buffer, (C) 70% DMSO-*d*_6_: 30% pH 5 sodium acetate buffer, (D) 65% DMSO-*d*_6_: 35% pH 5 sodium acetate buffer, (E) 60% DMSO-*d*_6_: 40% pH 5 sodium acetate buffer, (F) 55% DMSO-*d*_6_: 45% pH 5 sodium acetate buffer, (G) 50% DMSO-*d*_6_: 50% pH 5 sodium acetate buffer, (H) 45% DMSO-d_6_: 55% pH 5 sodium acetate buffer, (I) 40% DMSO-*d*_6_: 60% pH 5 sodium acetate buffer, (J) 35% DMSO-*d*_6_: 65% pH 5 sodium acetate buffer, (K) 30% DMSO-*d*_6_: 70% pH 5 sodium acetate buffer, (L) 25% DMSO-*d*_6_: 75% pH 5 sodium acetate buffer. The concentration of both S-MGB **4** and **10** were fixed at 1.5 mM in each experiment. All ^1^H-NMR spectral were recorded using a Bruker AVIIIHD nanobay instrument @ 400 MHz and TopSpin 3.6.2, using the solvent system compositions specified. Each ^1^H-NMR experiment was run with 32 scans.

## Author contributions

DPB, LMCM and CI carried out the NMR aggregation studies. FG, KG, CDS and FJS carried out the biological assays. FJS, JMC and AIK carried out the chemical synthesis. KCC, MPB, CJS and FJS conceived, designed and supervised the studies. FJS, CJS, DPB, LMCM and FG prepared the manuscript. All authors contributed to proofing the manuscript.

## Conflicts of interest

There is no conflict of interest to declare.

## Supplementary Material

MD-012-D1MD00110H-s001
