# Correction: Truncated S-MGBs: towards a parasite-specific and low aggregation chemotype

**DOI:** 10.1039/d1md90044g

**Published:** 2021-11-26

**Authors:** Daniel P. Brooke, Leah M. C. McGee, Federica Giordani, Jasmine M. Cross, Abedawn I. Khalaf, Craig Irving, Kirsten Gillingwater, Craig D. Shaw, Katharine C. Carter, Michael P. Barrett, Colin J. Suckling, Fraser J. Scott

**Affiliations:** WestCHEM Department of Pure and Applied Chemistry, University of Strathclyde Glasgow UK fraser.j.scott@strath.ac.uk; Wellcome Centre for Integrative Parasitology, Institute of Infection, Immunity and Inflammation and Glasgow Polyomics, College of Medical, Veterinary and Life Sciences, University of Glasgow Glasgow UK; Parasite Chemotherapy Unit, Department of Medical Parasitology and Infection Biology, Swiss Tropical and Public Health Institute Basel Switzerland; University of Basel Basel Switzerland; Strathclyde Institute of Pharmacy and Biomedical Science, University of Strathclyde Glasgow UK

## Abstract

Correction for ‘Truncated S-MGBs: towards a parasite-specific and low aggregation chemotype’ by Daniel P. Brooke *et al.*, *RSC Med. Chem.*, 2021, **12**, 1391–1401, DOI: 10.1039/D1MD00110H.

The authors regret that they omitted to include the following statement at the end of the Acknowledgements section: this work is based on research funded in part by the Bill & Melinda Gates Foundation (Investment ID OPP1093639) and with UK aid from the UK Government (Project 300504) through GALVmed. The findings and conclusions contained within are those of the authors and do not necessarily reflect positions or policies of the Bill & Melinda Gates Foundation or the UK Government.

The Royal Society of Chemistry apologises for these errors and any consequent inconvenience to authors and readers.

## Supplementary Material

