# Supplementary material for: Truncated S-MGBs: towards a parasite-specific and low aggregation chemotype
Source: RSC Med Chem. 2021 Jul 6;12(8):1391–401. doi: 10.1039/d1md00110h (PMC8372214; doi:10.1039/d1md00110h)
Supplement: MD-012-D1MD00110H-s001 [file MD-012-D1MD00110H-s001.pdf]

Electronic Supplementary Material (ESI) for RSC Medicinal Chemistry.
This journal is © The Royal Society of Chemistry 2021

Truncated S-MGBs: Towards a parasite-specific and low aggregation chemotype, ESI

Contents Page

Experimental Data of ^{1}HNMR investigations of S-MGB **4** 2

^{1}HNMR analysis and data of S-MGB **4** 2

^{1}HNMR solvent composition experiments of S-MGB **4** 3

^{1}HNMR proton peak shift analysis of compound S-MGB **4** 4

Experimental Data of ^{1}HNMR investigations of S-MGB **10** 9

^{1}HNMR analysis and data of S-MGB **10** 9

^{1}HNMR solvent composition experiments of S-MGB **10.** 10

^{1}HNMR proton peak shift analysis of compound S-MGB **10** 11

Acquired 2D DOSY and diffusion data for S-MGB **4** and S-MGB **10** 14

2D DOSY spectra MGB **4** 14

Accompanying diffusion data for S-MGB 4 15

Acquired 2D DOSY and diffusion data for S-MGB **10** 16

2D DOSY spectra S-MGB **10** 16

Accompanying diffusion data for S-MGB 10 16

2D DOSY spectra of S-MGB **4** and S-MGB **10** overlay 17

Experimental Data of ^{1}HNMR investigations of S-MGB **4**

^{1}HNMR analysis and data of S-MGB **4**

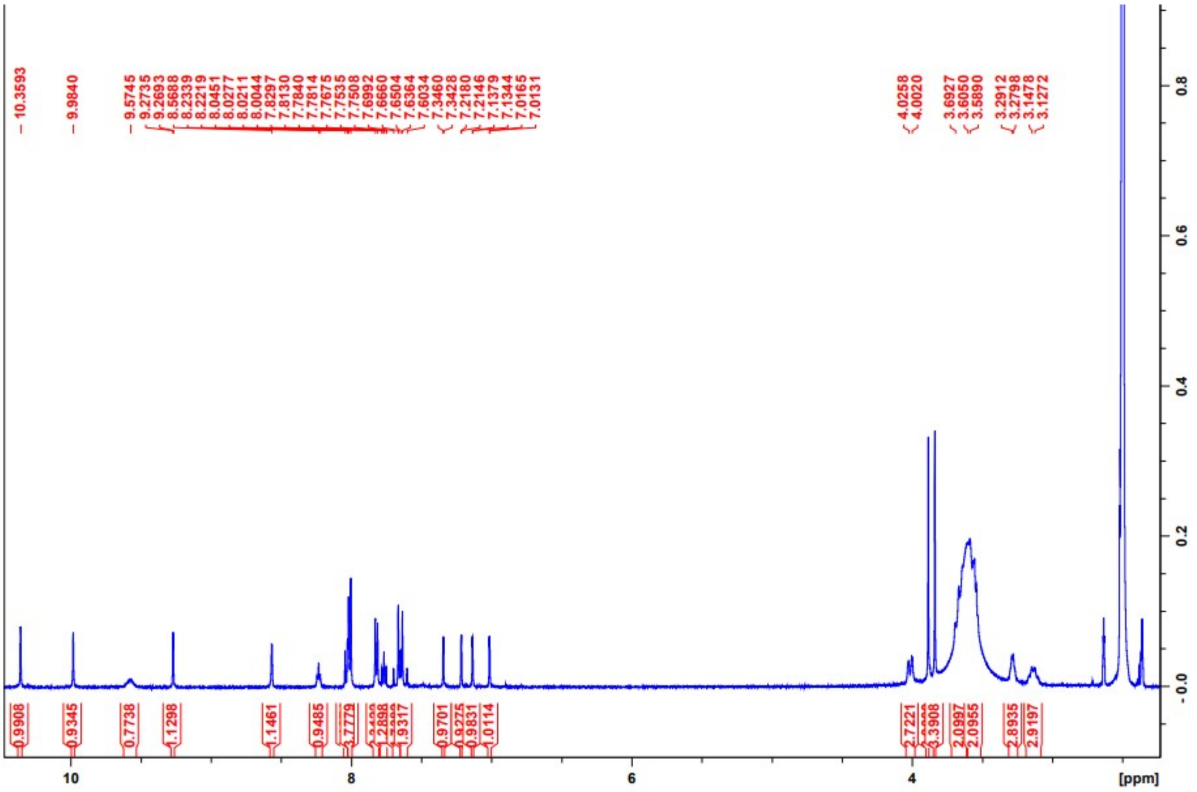

Figure S1. ^{1}H NMR spectra of **4.** Solvent composition 100% DMSO-d_6

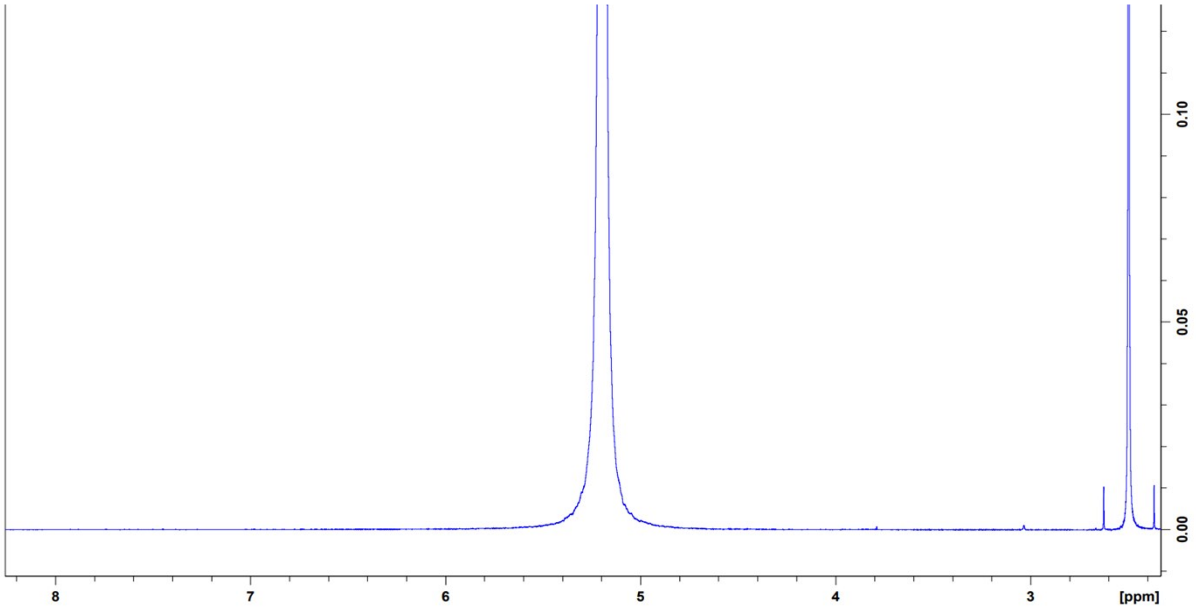

Figure S2. ^{1}H NMR spectra of **4.** Solvent composition 100% 0.1M sodium acetate buffer.

^{1}HNMR solvent composition experiments of S-MGB **4**

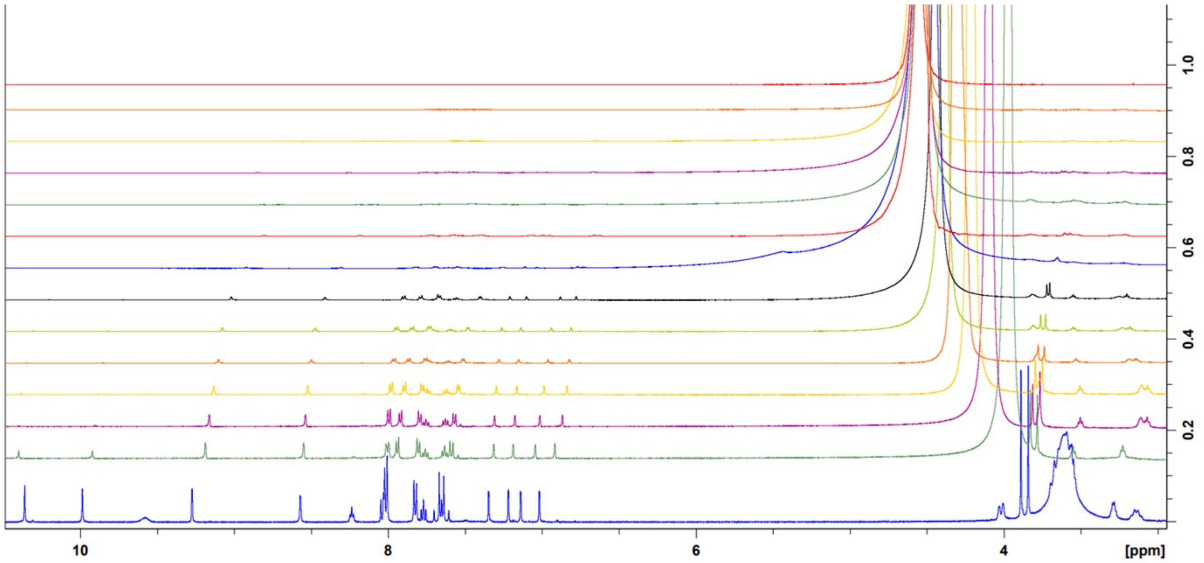

Figure S3. Full overlaid ^{1}H NMR of compound **4** composition experiment. The spectra were obtained with the following solvent system compositions: (A) 100 % DMSO-d_6 (B) 80 % DMSO-d_6: 20 % 0.1M pH5 sodium acetate buffer, (C) 75 % DMSO-d_6: 25 % 0.1 M pH5 sodium acetate buffer, (D) 70 % DMSO-d_6: 30 % 0.1 M pH5 sodium acetate buffer, (E) 65 % DMSO-d_6: 35 % 0.1M pH5 sodium acetate buffer, (F) 60 % DMSO-d_6: 40 % 0.1M pH5 sodium acetate buffer, (G) 55 % DMSO-d_6: 45 % 0.1 M pH5 sodium acetate buffer, (H) 50 % DMSO-d_6: 50 % 0.1M pH5 sodium acetate buffer, (I) 45 % DMSO-d_6: 55 % 0.1M pH5 sodium acetate buffer, (J) 40 % DMSO-d_6: 60 % 0.1M pH5 sodium acetate buffer, (K) 35 % DMSO-d_6: 65 % 0.1M pH5 sodium acetate buffer, (L) 30 % DMSO-d_6: 70 % 0.1M pH5 sodium acetate buffer, (M) 25 % DMSO-d_6: 75 % 0.1M pH5 sodium acetate buffer, (N) 100 % 0.1M pH5 sodium acetate buffer The concentration of S-MGB **4** was fixed at 1.5 mM in each experiment.

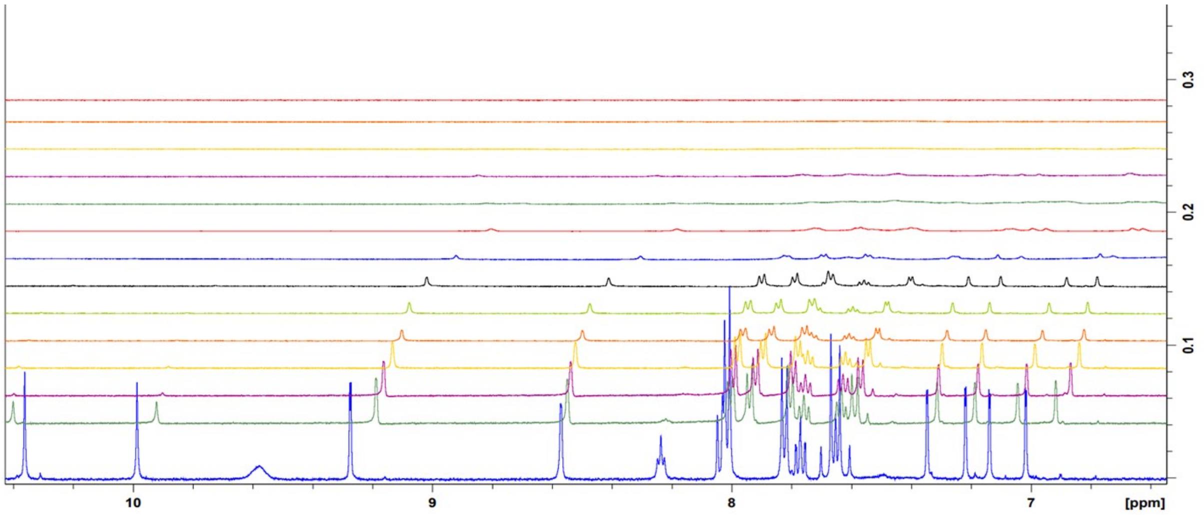

Figure S4. ^{1}H NMR of the aromatic region of compound **4** composition experiment. The spectra were obtained with the following solvent system compositions: (A) 100 % DMSO-d_6 (B) 80 % DMSO-d_6: 20 % 0.1M pH5 sodium acetate buffer, (C) 75 % DMSO-d_6: 25 % 0.1M pH5 sodium acetate buffer, (D) 70 % DMSO-d_6: 30 % 0.1M pH5 sodium acetate buffer, (E) 65 % DMSO-d_6: 35 % 0.1M pH5 sodium acetate buffer, (F) 60 % DMSO-d_6: 40 % 0.1M pH5 sodium acetate buffer, (G) 55 % DMSO-d_6: 45 % 0.1M pH5 sodium acetate buffer, (H) 50 % DMSO-d_6: 50 % 0.1M pH5 sodium acetate buffer, (I) 45 % DMSO-d_6: 55 % 0.1M pH5 sodium acetate buffer, (J) 40 % DMSO-d_6: 60 % 0.1M pH5 sodium acetate buffer, (K) 35 % DMSO-d_6: 65 % 0.1M pH5 sodium acetate buffer, (L) 30 % DMSO-d_6: 70 % 0.1M pH5 sodium acetate buffer, (M) 25 % DMSO-d_6: 75 % 0.1M pH5 sodium acetate buffer, (N) 100 % pH5 sodium acetate buffer The concentration of S-MGB **4** was fixed at 1.5 mM in each experiment.

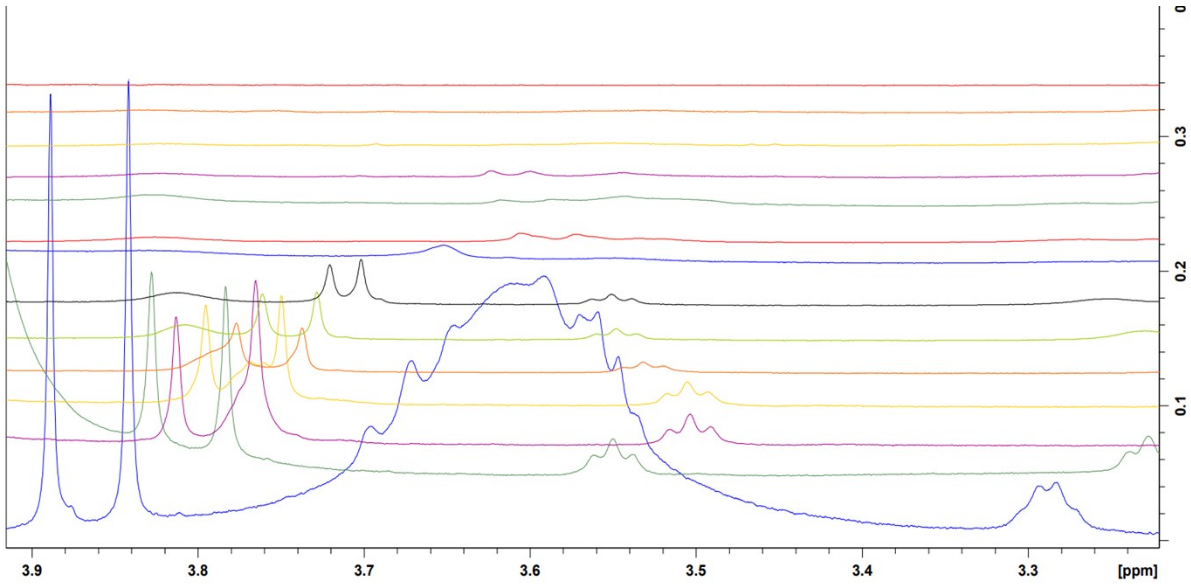

Figure S5. ^{1}H NMR of the aliphatic region of compound **4** composition experiment. The spectra were obtained with the following solvent system compositions: (A) 100 % DMSO-d_6 (B) 80 % DMSO-d_6: 20 % 0.1M pH5 sodium acetate buffer, (C) 75 % DMSO-d_6: 25 % 0.1M pH5 sodium acetate buffer, (D) 70 % DMSO-d_6: 30 % 0.1M pH5 sodium acetate buffer, (E) 65 % DMSO-d_6: 35 % 0.1M pH5 sodium acetate buffer, (F) 60 % DMSO-d_6: 40 % 0.1M pH5 sodium acetate buffer, (G) 55 % DMSO-d_6: 45 % 0.1M pH5 sodium acetate buffer, (H) 50 % DMSO-d_6: 50 % 0.1M pH5 sodium acetate buffer, (I) 45 % DMSO-d_6: 55 % 0.1M pH5 sodium acetate buffer, (J) 40 % DMSO-d_6: 60 % 0.1M pH5 sodium acetate buffer, (K) 35 % DMSO-d_6: 65 % 0.1M pH5 sodium acetate buffer, (L) 30 % DMSO-d_6: 70 % 0.1M pH5 sodium acetate buffer, (M) 25 % DMSO-d_6: 75 % 0.1M pH5 sodium acetate buffer, (N) 100 % pH5 sodium acetate buffer The concentration of S-MGB **4** was fixed at 1.5 mM in each experiment.

^{1}HNMR proton peak shift analysis of compound S-MGB **4**

^{1}H-NMR spectra were systematically acquired in solvent systems with different proportions of DMSO-d_6 and pH5 sodium acetate buffer, using a fixed concentration of 1.5mM of S-MGB. The graphs below display the data obtained, with each graph focusing on the ^{1}HNMR peak shift of a specific proton of compound S-MGB **4,** labelled in **Figure S6**.

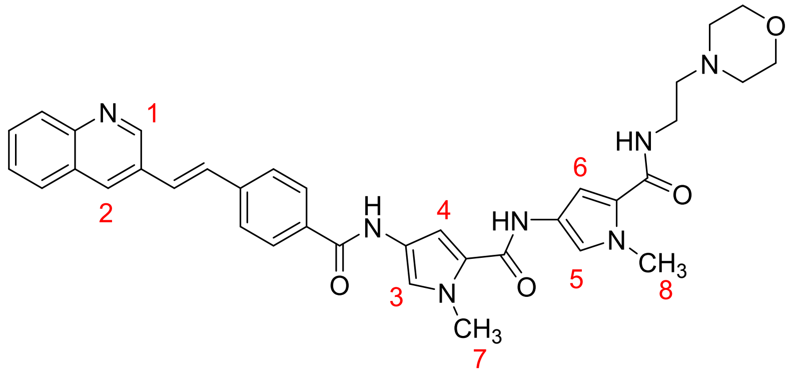

Figure S6. Structure and specific proton assignment of S-MGB **4**.

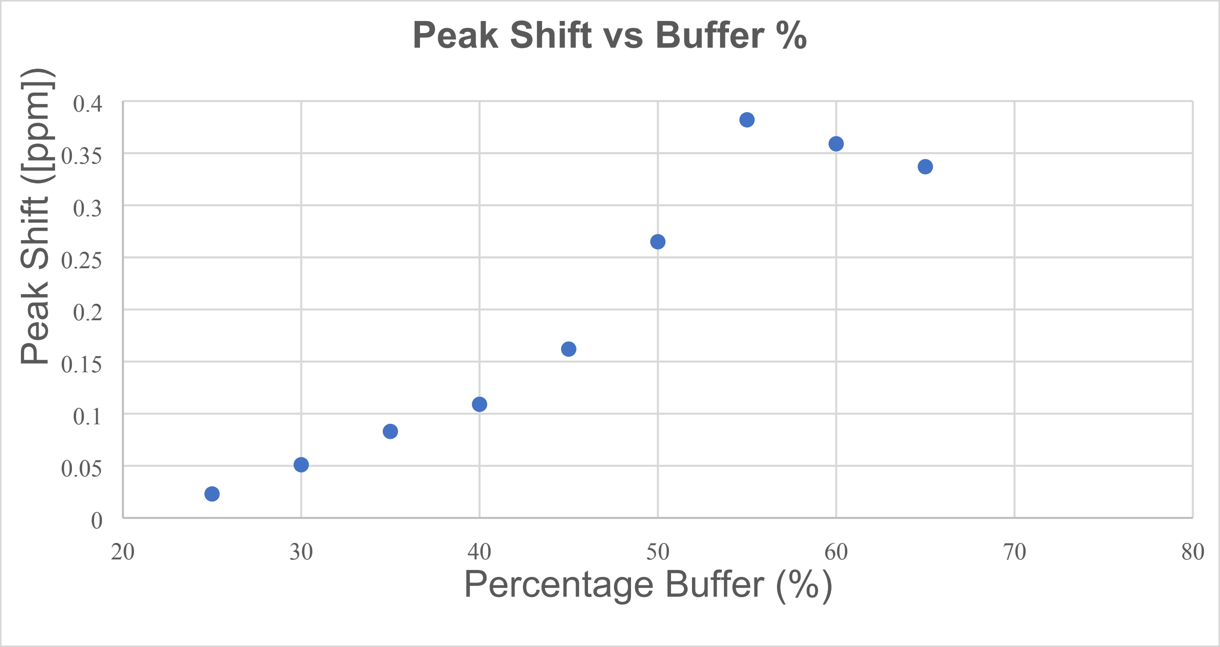

Graph S1. Peak shift difference of quinoline proton **1** in varying solvent system compositions.

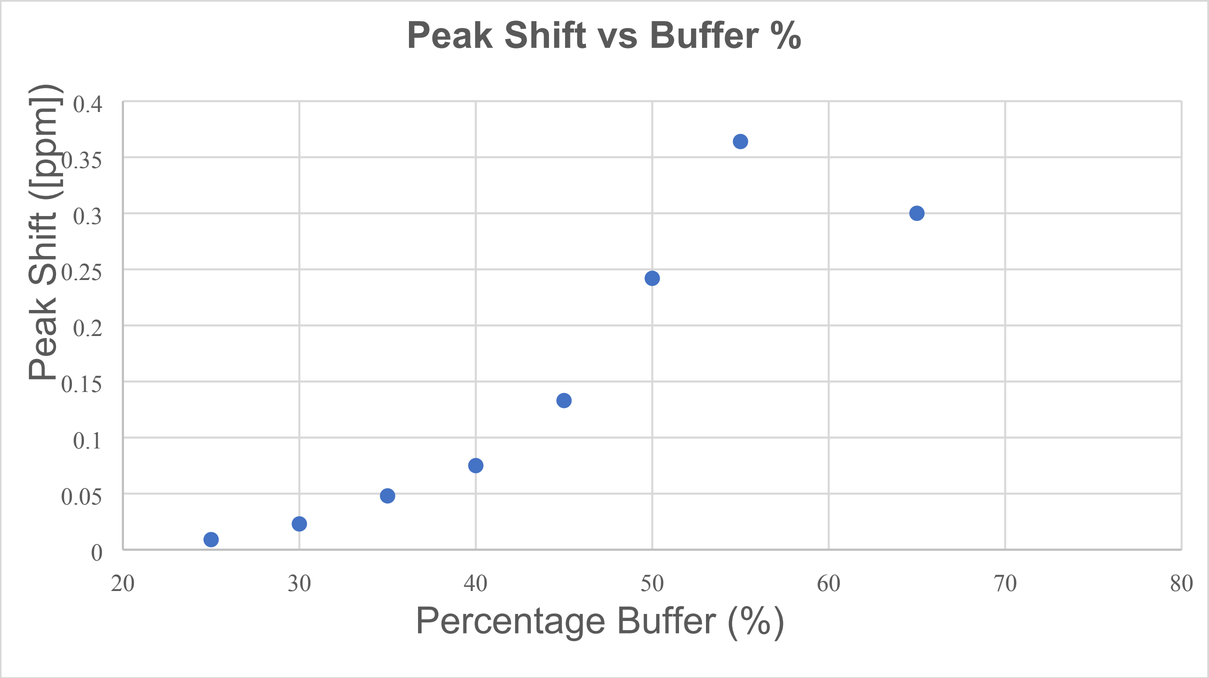

Graph S2. Peak shift difference of quinoline proton **2** in varying solvent system compositions.

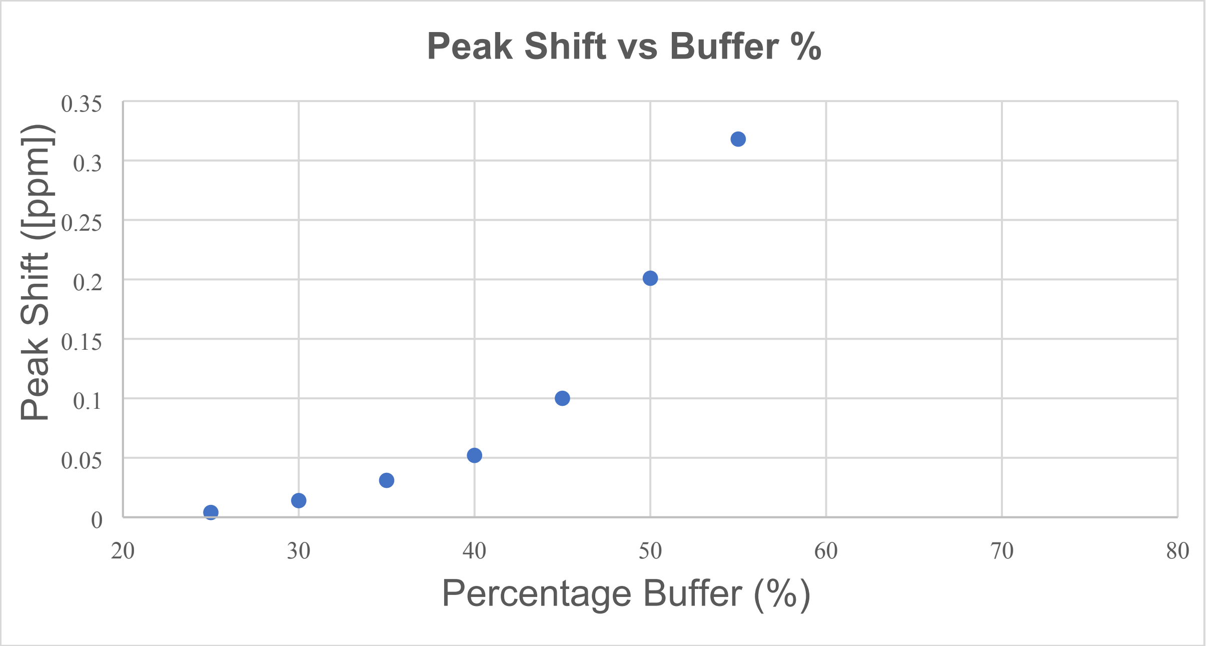

Graph S3. Peak shift difference of pyrrole proton **3** in varying solvent system compositions.

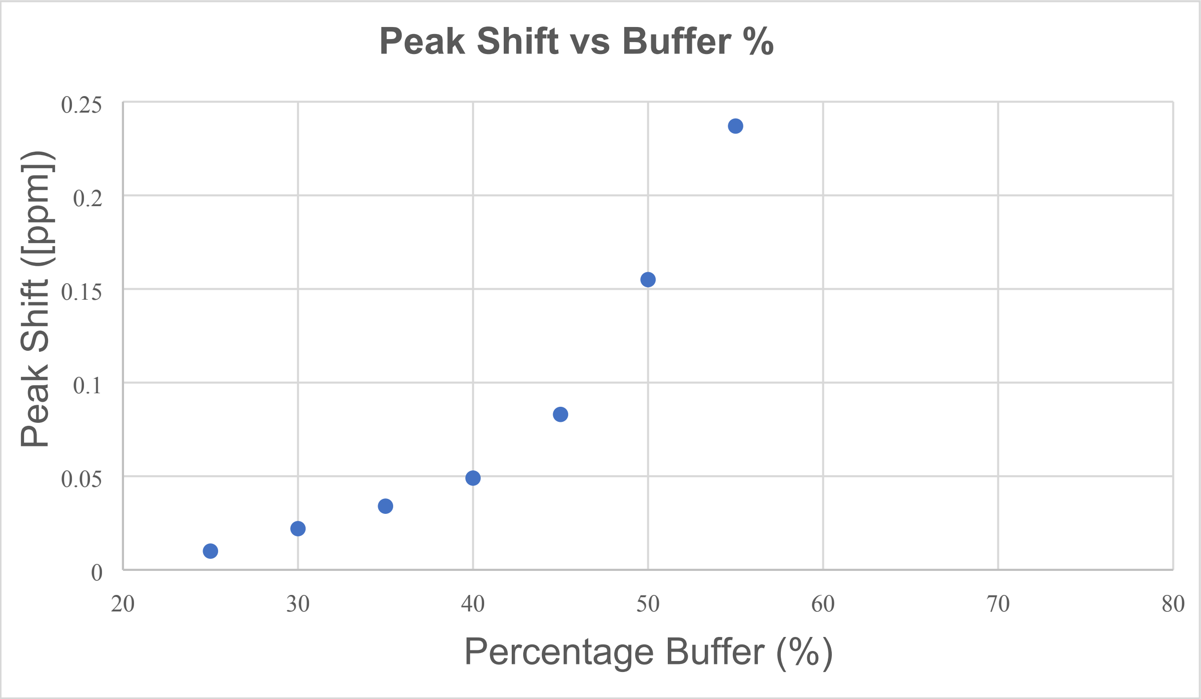

Graph S4. Peak shift difference of pyrrole proton **4** in varying solvent system compositions.

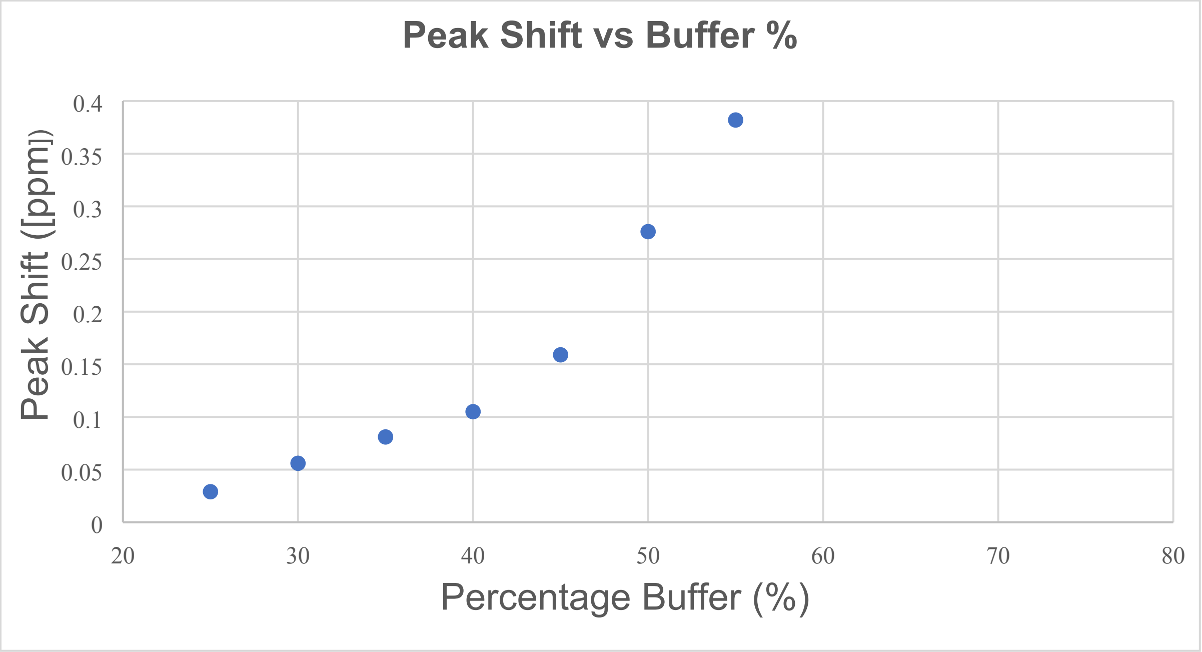

Graph S5. Peak shift difference of pyrrole proton **5** in varying solvent system compositions.

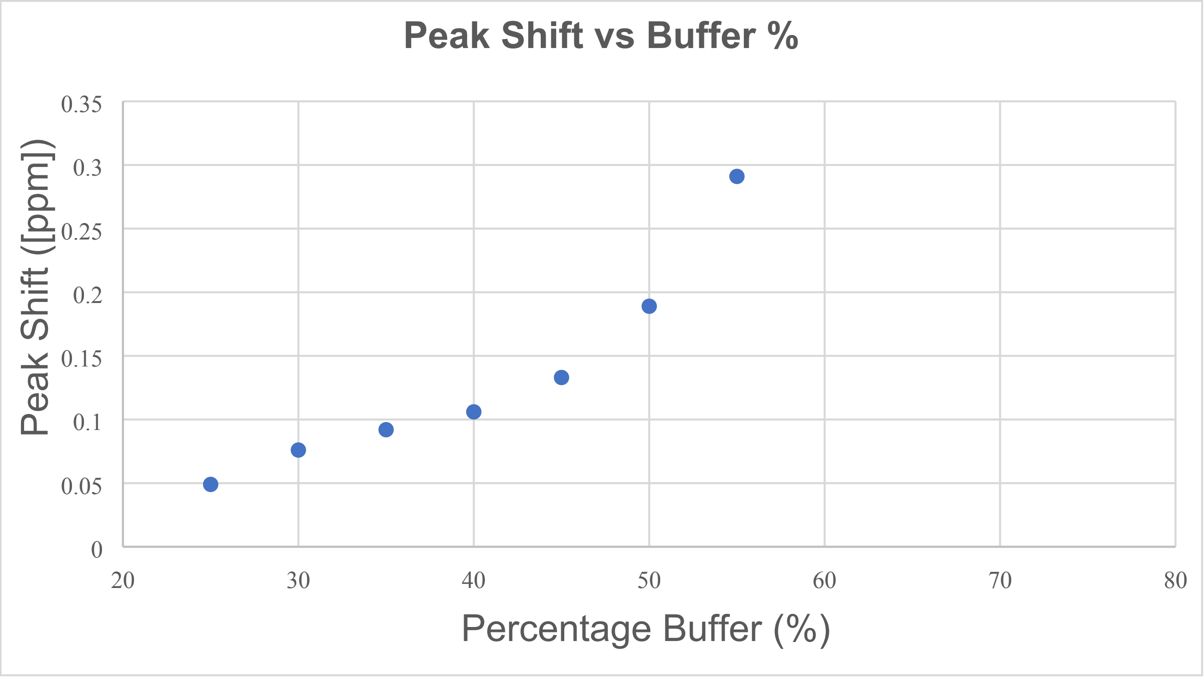

Graph S6. Peak shift difference of pyrrole proton **6** in varying solvent system compositions.

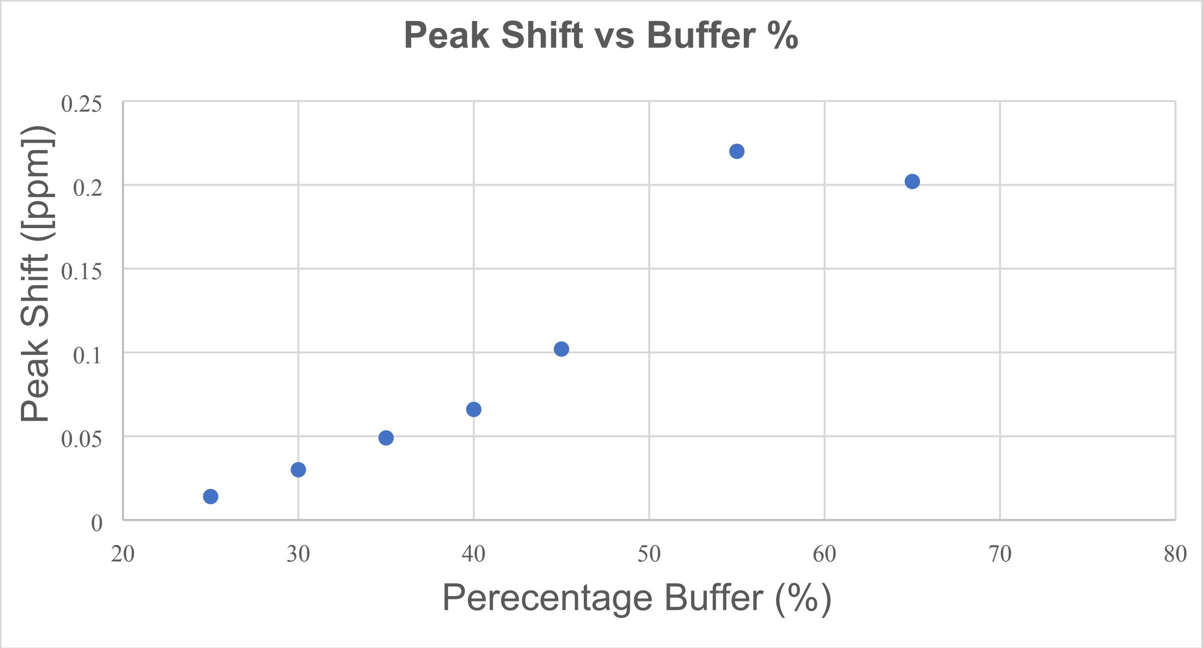

Graph S7. Peak shift difference of methyl proton **7** in varying solvent system compositions.

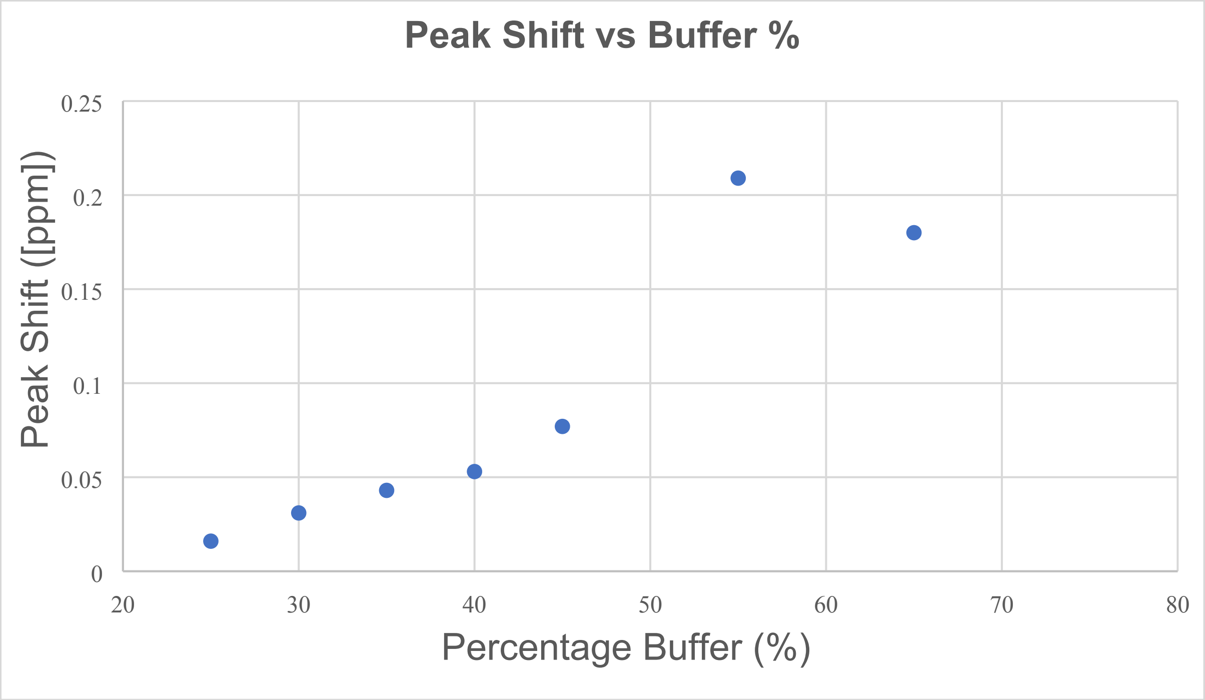

Graph S8. Peak shift difference of methyl proton **8** in varying solvent system compositions.

Experimental Data of ^{1}HNMR investigations of S-MGB **10**

^{1}HNMR analysis and data of S-MGB **10**

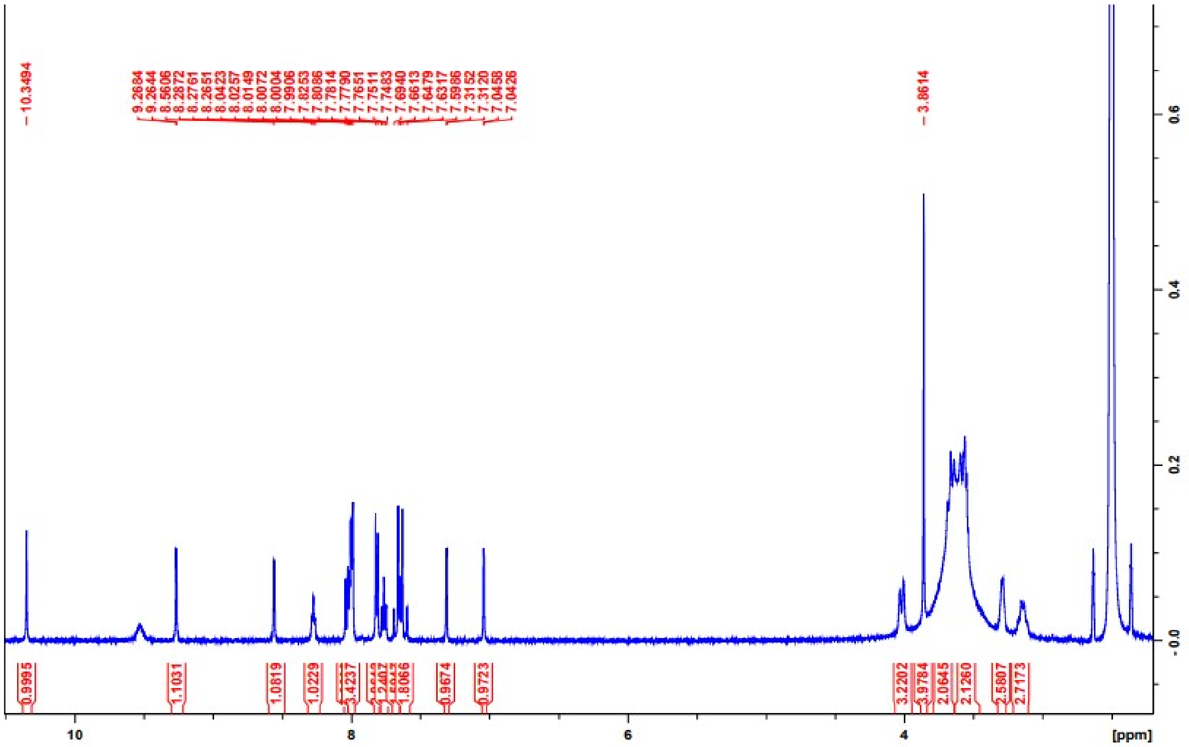

Figure S7. ^{1}H NMR spectra of **10.** Solvent composition 100% DMSO-d_6

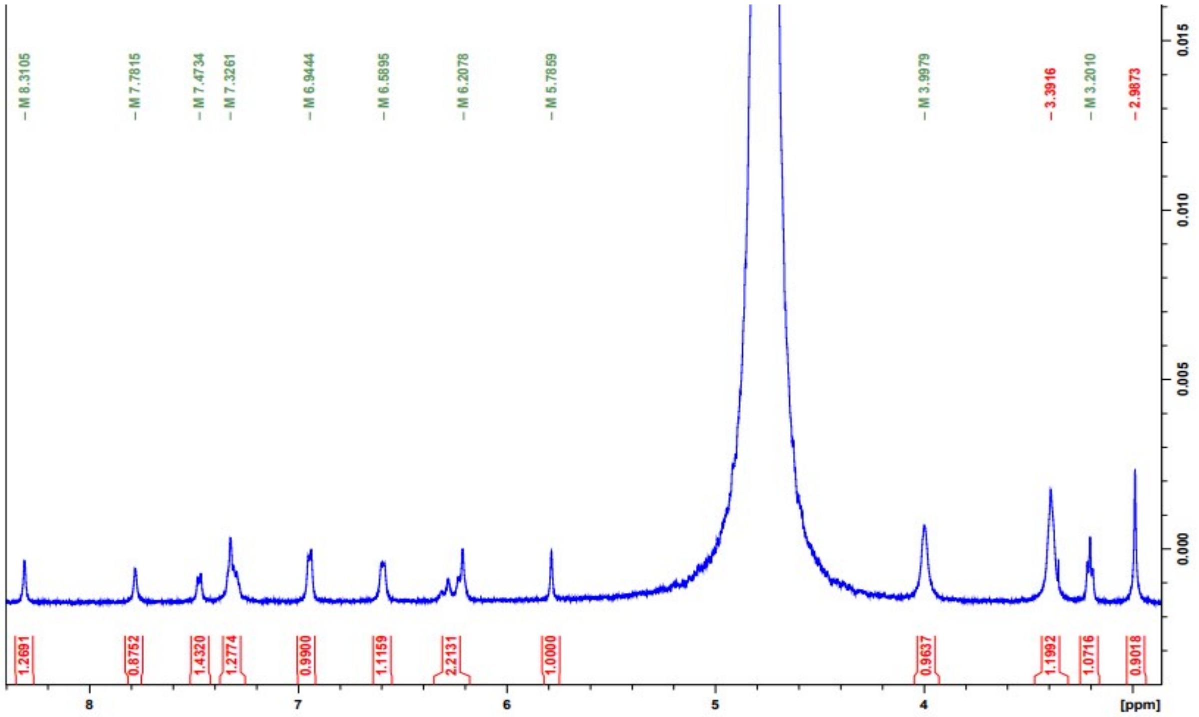

Figure S8. ^{1}H NMR spectra of **10.** Solvent composition 100% sodium acetate buffer.

^{1}HNMR solvent composition experiments of S-MGB **10.**

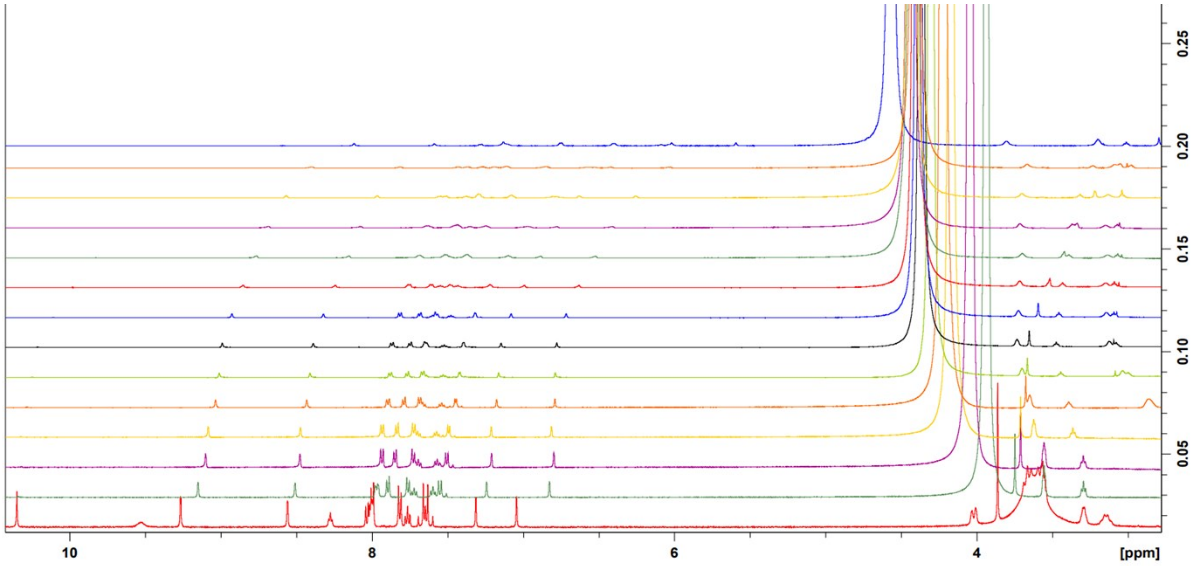

Figure S9. Full ^{1}H NMR spectra of compound **10** composition experiment. The spectra were obtained with the following solvent system compositions: (A) 100 % DMSO-d_6 (B) 80 % DMSO-d_6: 20 % 0.1M pH5 sodium acetate buffer, (C) 75 % DMSO-d_6: 25 % 0.1M pH5 sodium acetate buffer, (D) 70 % DMSO-d_6: 30 % 0.1M pH5 sodium acetate buffer, (E) 65 % DMSO-d_6: 35 % 0.1M pH5 sodium acetate buffer, (F) 60 % DMSO-d_6: 40 % 0.1M pH5 sodium acetate buffer, (G) 55 % DMSO-d_6: 45 % 0.1M pH5 sodium acetate buffer, (H) 50 % DMSO-d_6: 50 % 0.1M pH5 sodium acetate buffer, (I) 45 % DMSO-d_6: 55 % 0.1M pH5 sodium acetate buffer, (J) 40 % DMSO-d_6: 60 % 0.1M pH5 sodium acetate buffer, (K) 35 % DMSO-d_6: 65 % 0.1M pH5 sodium acetate buffer, (L) 30 % DMSO-d_6: 70 % 0.1M pH5 sodium acetate buffer, (M) 25 % DMSO-d_6: 75 % 0.1M pH5 sodium acetate buffer, (N) 100 % pH5 sodium acetate buffer The concentration of S-MGB **10** was fixed at 1.5 mM in each experiment.

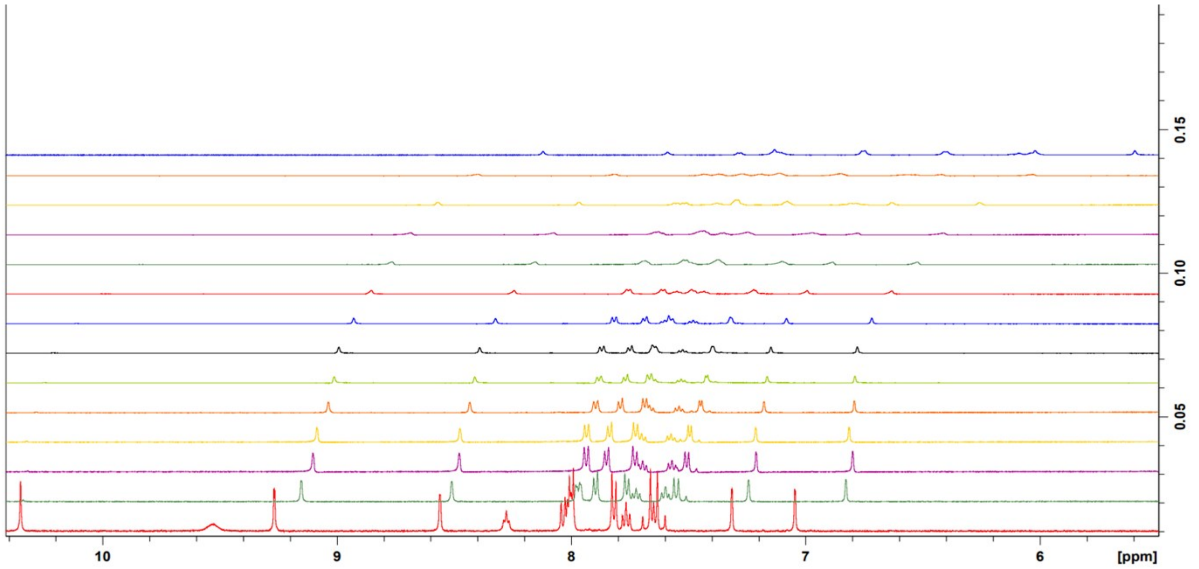

Figure S10. ^{1}H NMR spectra of the aromatic region of compound **10** composition experiment. The spectra were obtained with the following solvent system compositions: (A) 100 % DMSO-d_6 (B) 80 % DMSO-d_6: 20 % 0.1M pH5 sodium acetate buffer, (C) 75 % DMSO-d_6: 25 % 0.1M pH5 sodium acetate buffer, (D) 70 % DMSO-d_6: 30 % 0.1M pH5 sodium acetate buffer, (E) 65 % DMSO-d_6: 35 % 0.1M pH5 sodium acetate buffer, (F) 60 % DMSO-d_6: 40 % 0.1M pH5 sodium acetate buffer, (G) 55 % DMSO-d_6: 45 % 0.1M pH5 sodium acetate buffer, (H) 50 % DMSO-d_6: 50 % 0.1M pH5 sodium acetate buffer, (I) 45 % DMSO-d_6: 55 % 0.1M pH5 sodium acetate buffer, (J) 40 % DMSO-d_6: 60 % 0.1M pH5 sodium acetate buffer, (K) 35 % DMSO-d_6: 65 % 0.1M pH5 sodium acetate buffer, (L) 30 % DMSO-d_6: 70 % 0.1M pH5 sodium acetate buffer, (M) 25 % DMSO-d_6: 75 % 0.1M pH5 sodium acetate buffer, (N) 100 % pH5 sodium acetate buffer The concentration of S-MGB **10**was fixed at 1.5 mM in each experiment.

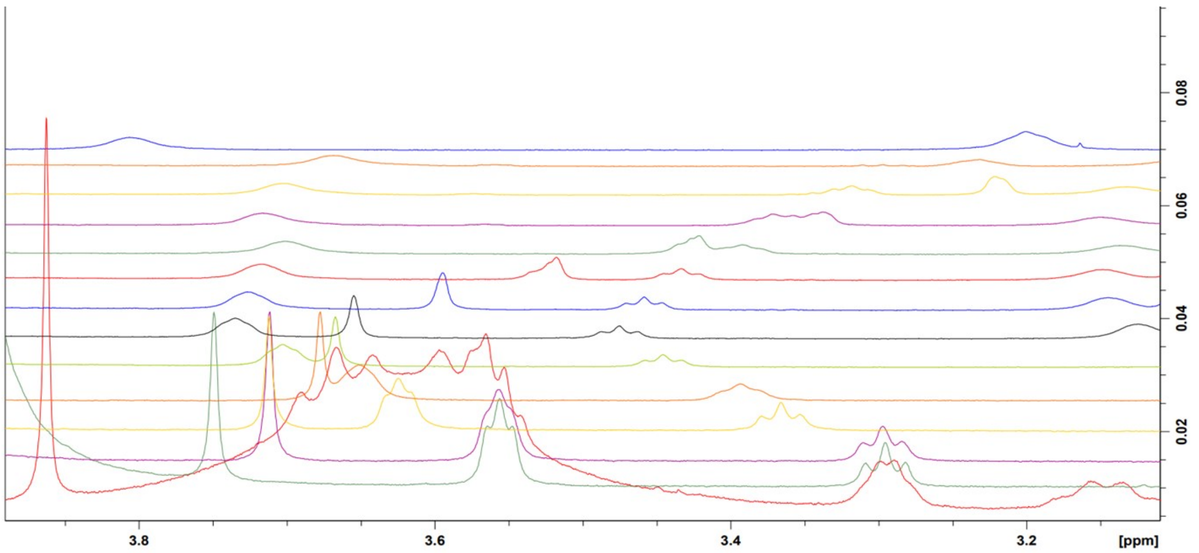

Figure S11. ^{1}H NMR spectra of aliphatic region of compound **10** composition experiment. The spectra were obtained with the following solvent system compositions: (A) 100 % DMSO-d_6 (B) 80 % DMSO-d_6: 20 % 0.1M pH5 sodium acetate buffer, (C) 75 % DMSO-d_6: 25 % 0.1M pH5 sodium acetate buffer, (D) 70 % DMSO-d_6: 30 % 0.1M pH5 sodium acetate buffer, (E) 65 % DMSO-d_6: 35 % 0.1M pH5 sodium acetate buffer, (F) 60 % DMSO-d_6: 40 % 0.1M pH5 sodium acetate buffer, (G) 55 % DMSO-d_6: 45 % 0.1M pH5 sodium acetate buffer, (H) 50 % DMSO-d_6: 50 % 0.1M pH5 sodium acetate buffer, (I) 45 % DMSO-d_6: 55 % 0.1M pH5 sodium acetate buffer, (J) 40 % DMSO-d_6: 60 % 0.1M pH5 sodium acetate buffer, (K) 35 % DMSO-d_6: 65 % 0.1M pH5 sodium acetate buffer, (L) 30 % DMSO-d_6: 70 % 0.1M pH5 sodium acetate buffer, (M) 25 % DMSO-d_6: 75 % 0.1M pH5 sodium acetate buffer, (N) 100 % pH5 sodium acetate buffer The concentration of S-MGB **10** was fixed at 1.5 mM in each experiment.

^{1}HNMR proton peak shift analysis of compound S-MGB **10**

^{1}H-NMR spectra were systematically acquired in solvent systems with different proportions of DMSO-d_6 and 0.1M pH5 sodium acetate buffer, using a fixed concentration of 1.5mM of S-MGB. The graphs below display the data obtained, with each graph focusing on the ^{1}HNMR peak shift of a specific proton of compound S-MGB **10,** labelled in **Figure S12**.

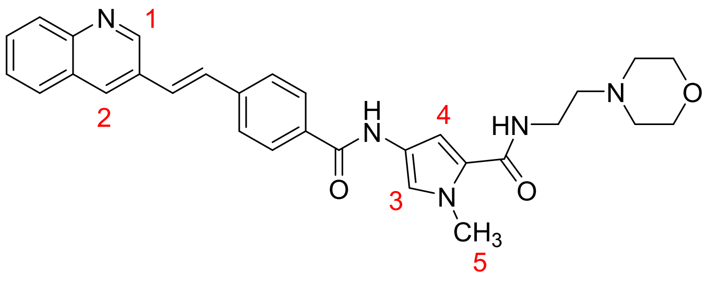

Figure S12. Structure and specific proton assignment of S-MGB **10**.

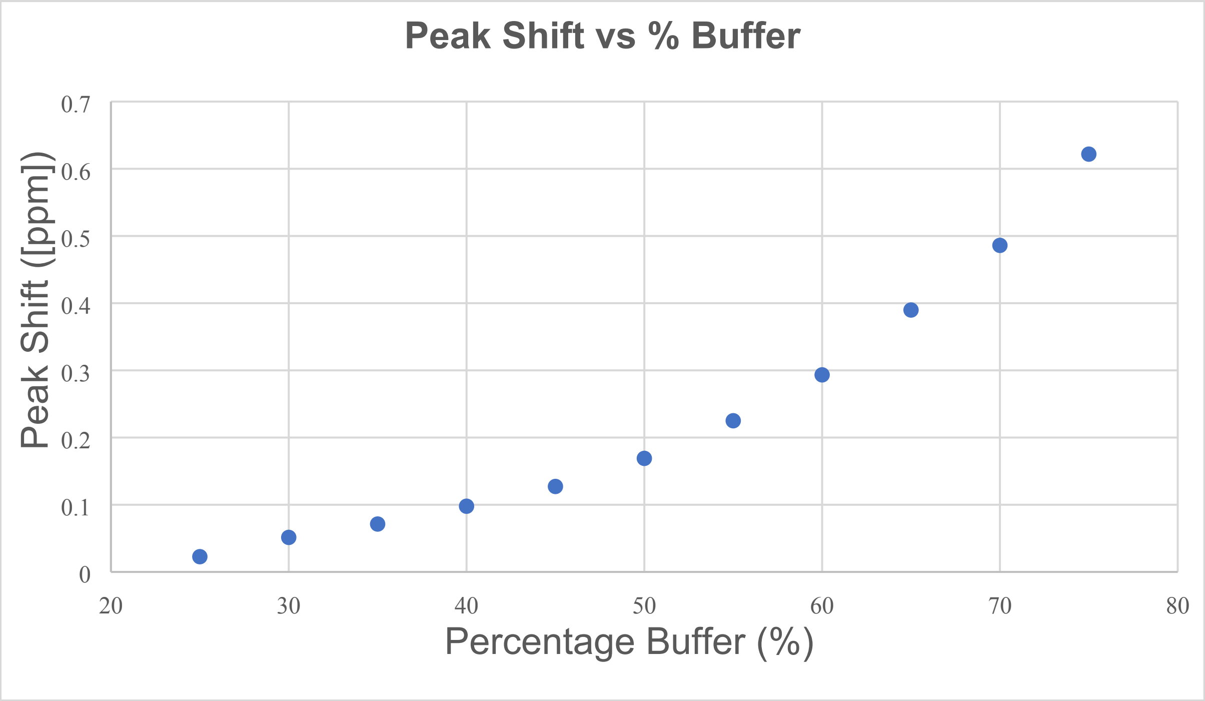

Graph S9. Peak shift difference of quinoline proton **1** in varying solvent system compositions.

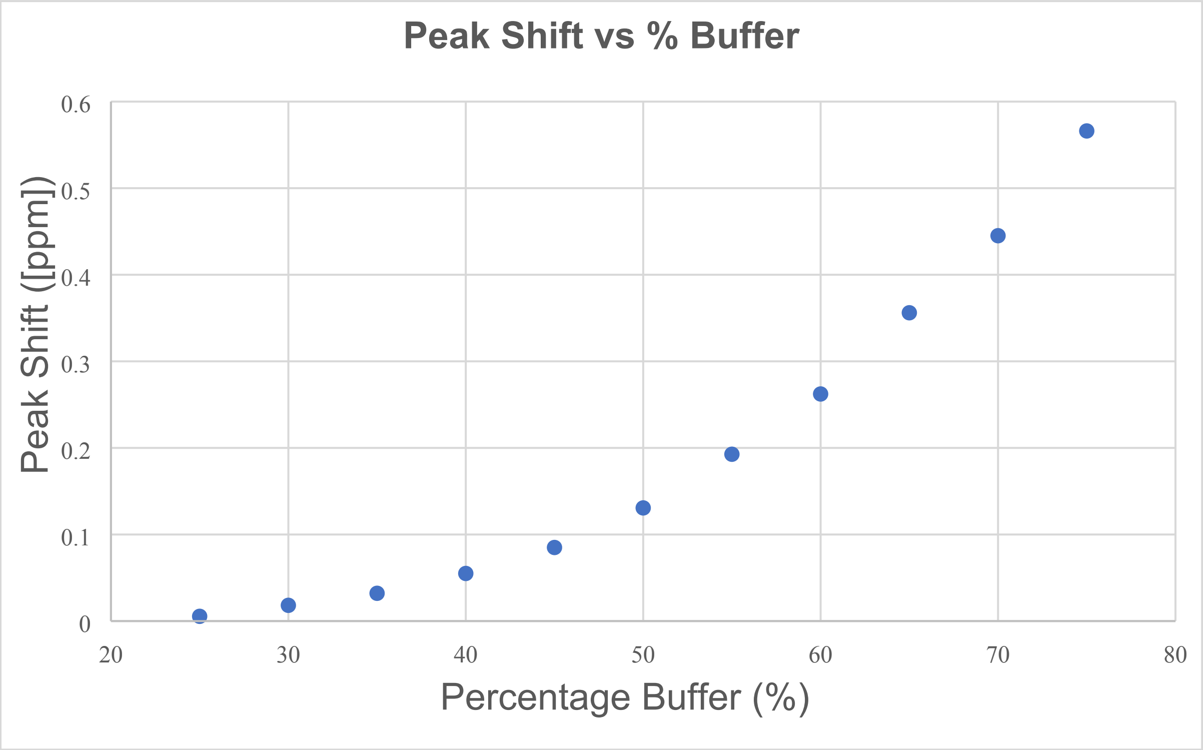

Graph S10. Peak shift difference of quinoline proton **2** in varying solvent system compositions.

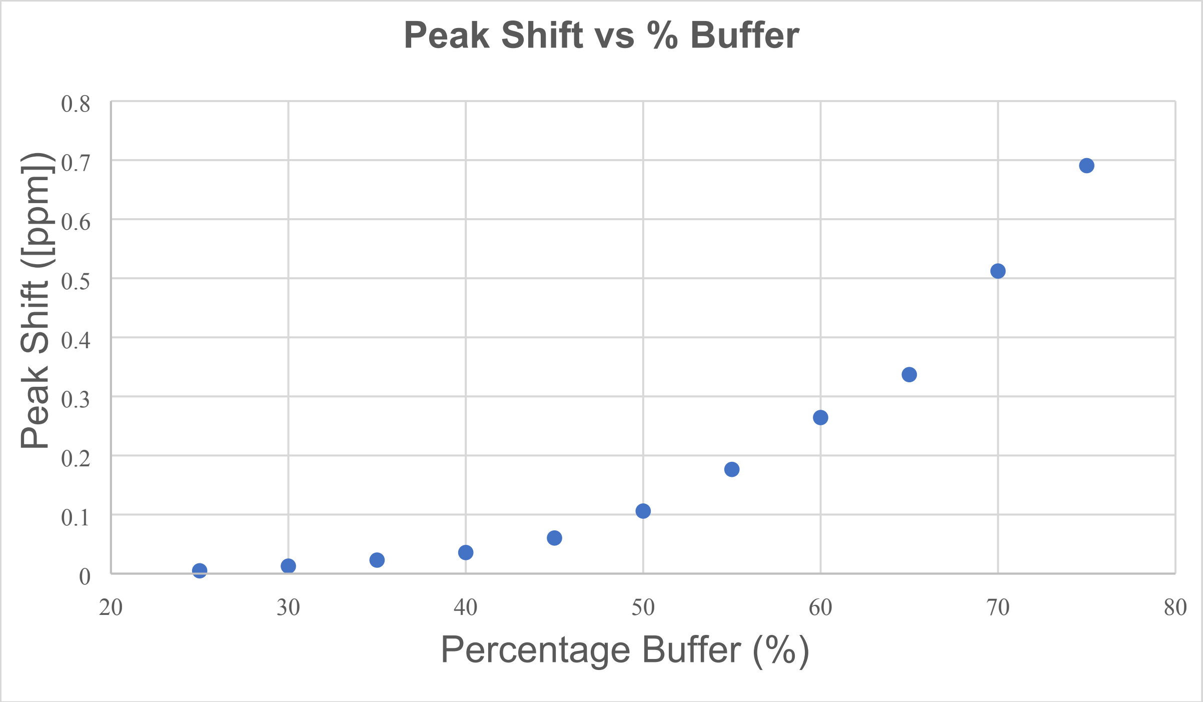

Graph S11. Peak shift difference of pyrrole proton **3** in varying solvent system compositions.

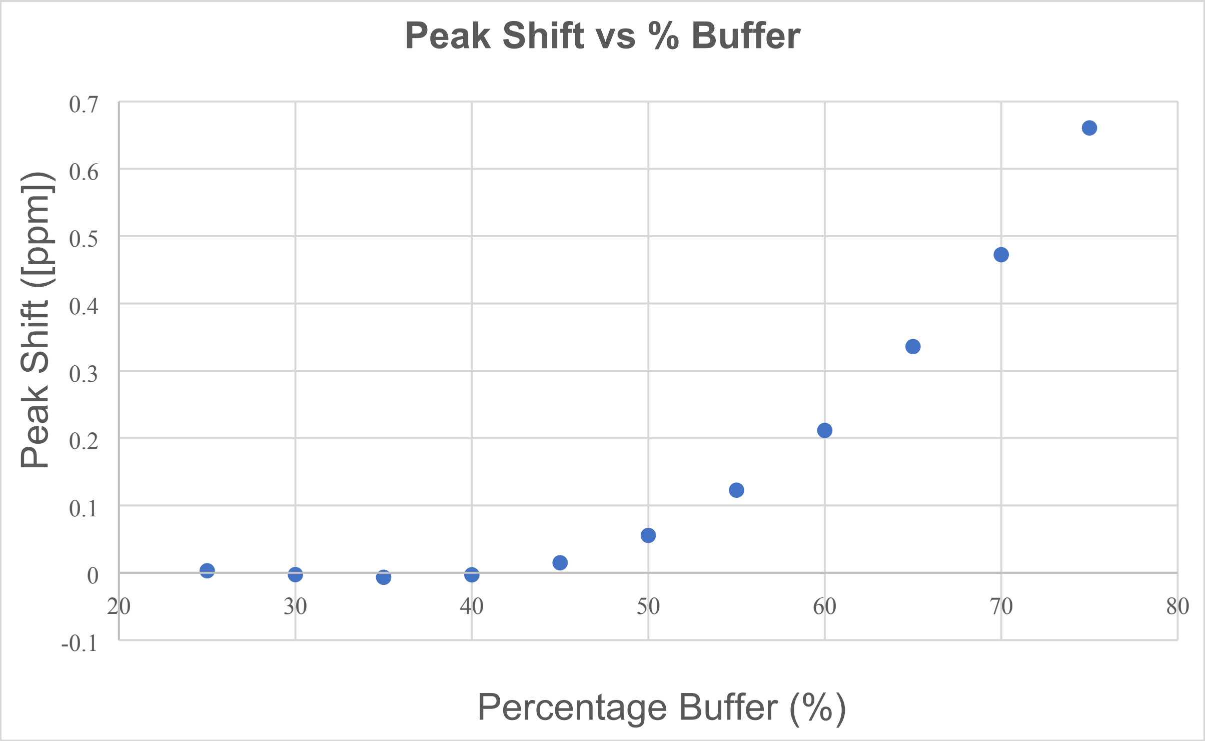

Graph S12. Peak shift difference of pyrrole proton **4** in varying solvent system compositions.

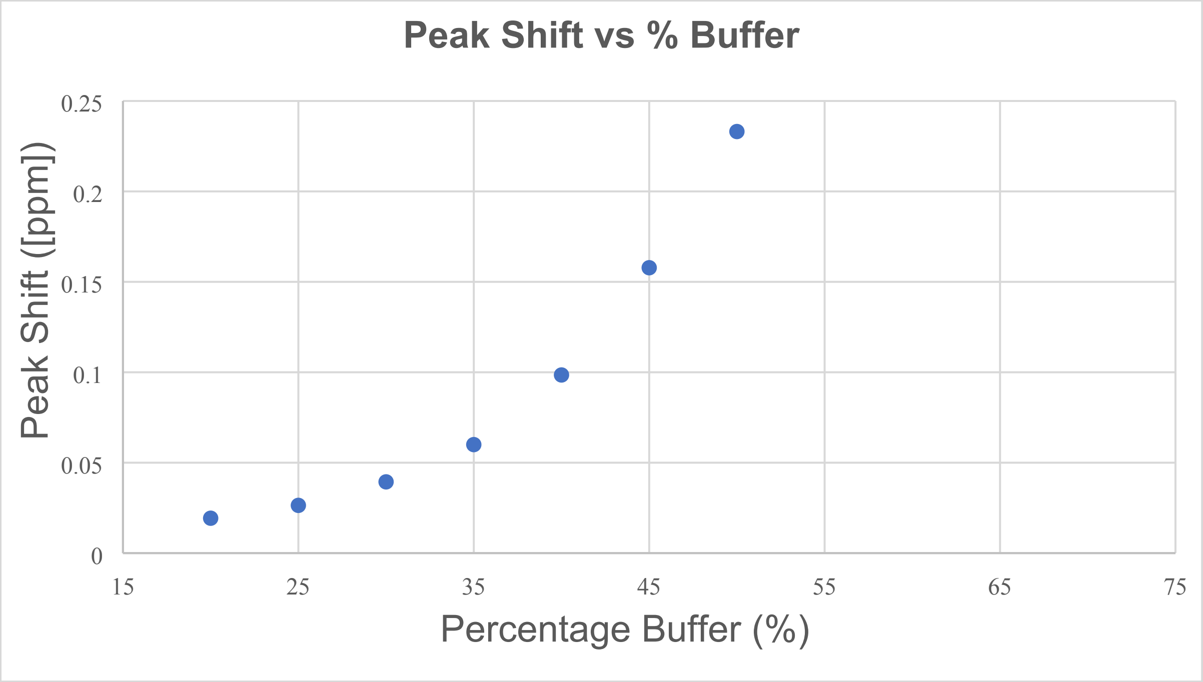

Graph S13. Peak shift difference of methyl proton **5** in varying solvent system compositions.

Acquired 2D DOSY and diffusion data for S-MGB **4** and S-MGB **10**

2D DOSY spectra MGB **4**

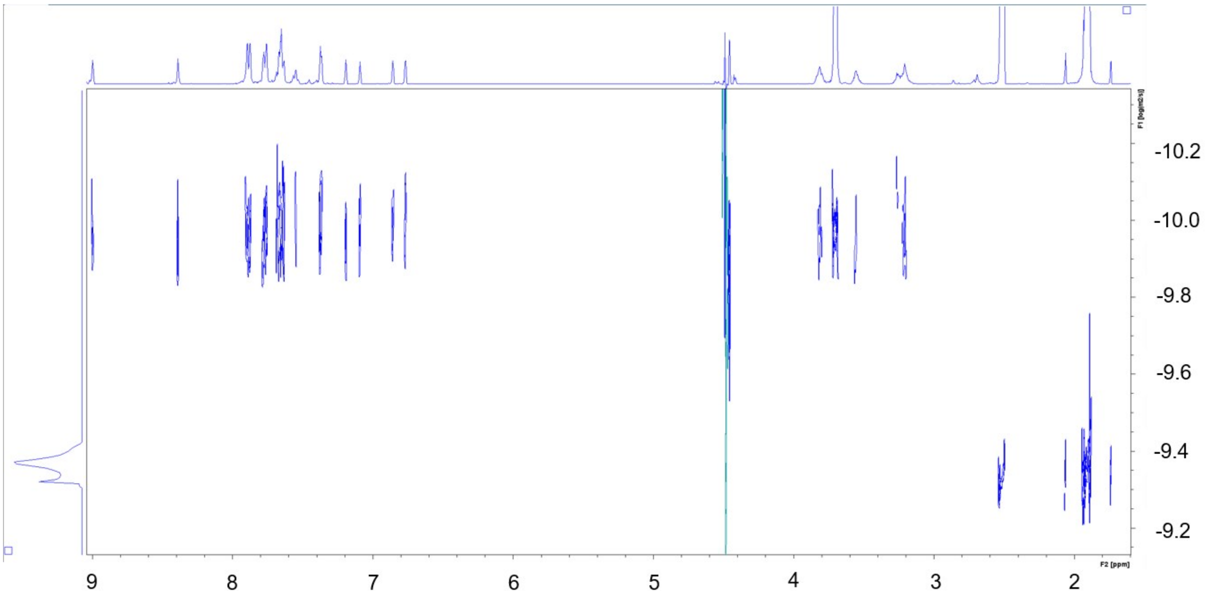

Figure S13. 2D DOSY NMR of **4.** Solvent system 50 % DMSO-d_6: 50 % 0.1M pH5 sodium acetate buffer.

Accompanying diffusion data for S-MGB **4**

Table S1. List of diffusion constants of **4** with the respective peak range.

**Peak Range (ppm)**	**Diffusion Constant ($m^2 s^{-1}$)**
9.008 – 8.984	1.069 $x10^{-10}$
8.396 – 8.378	1.127 $x10^{-10}$
7.903 – 7.863	1.099 $x10^{-10}$
7.782 – 7.748	1.121 $x10^{-10}$
7.691 – 7.624	1.031 $x10^{-10}$
7.575 – 7.523	1.021 $x10^{-10}$
7.386 – 7.355	9.948 $x10^{-11}$
7.205 – 7.182	1.183 $x10^{-10}$
7.099 – 7.081	1.051 $x10^{-10}$
6.867 – 6.847	9.919 $x10^{-11}$
6.779 – 6.756	9.769 $x10^{-11}$
3.843 – 3.688	1.118 $x10^{-10}$
3.725 – 3.688	1.051 $x10^{-10}$
3.299 – 3.160	1.115 $x10^{-10}$

Average – 1.068 $x10^{-10}$ $m^2 s^{-1}$

Standard Deviation - 0.061

Acquired 2D DOSY and diffusion data for S-MGB **10**

2D DOSY spectra S-MGB **10**

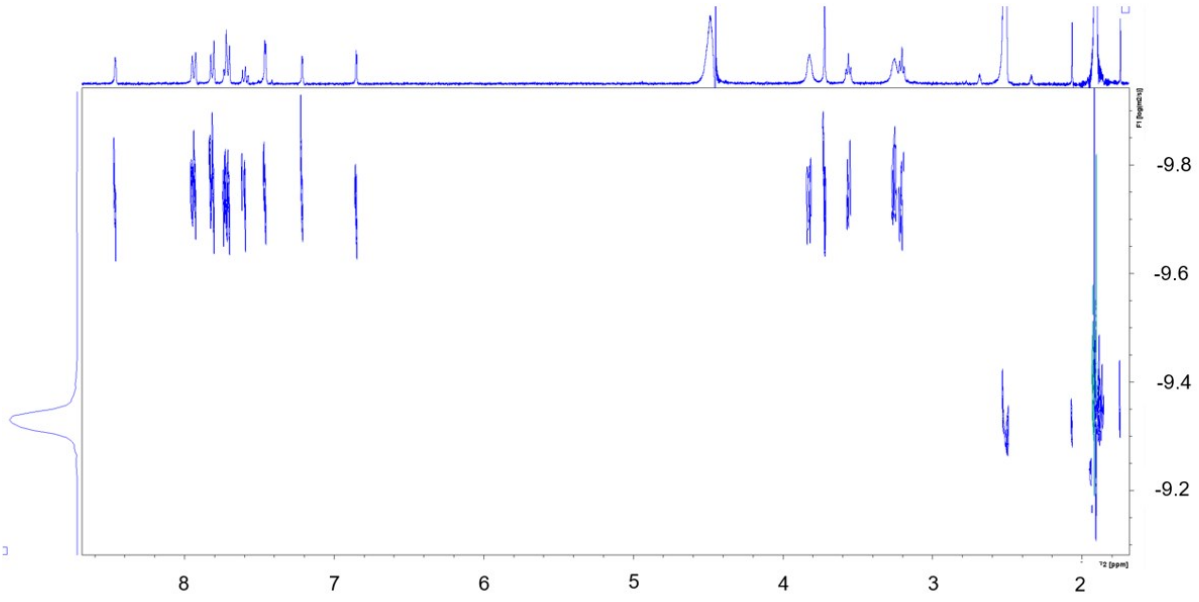

Figure S 14. 2D DOSY NMR of **10.** Solvent system 50 % DMSO-d_6: 50 % 0.1M pH5 sodium acetate buffer.

Accompanying diffusion data for S-MGB **10**

Table S2. List of diffusion constants of **10** with the respective peak range.

**Peak Range (ppm)**	**Diffusion Constant (m^2s^{-1})**
9.077 – 9.048	1.721 x10^{-10}
8.477 – 8.450	1.820 x10^{-10}
7.963 – 7.924	1.717 x10^{-10}
7.836 – 7.800	1.711 x10^{-10}
7.732 – 7.696	1.806 x10^{-10}
7.623 – 7.572	1.670 x10^{-10}
7.477 – 7.451	1.748 x10^{-10}
7.227 – 7.206	1.731 x10^{-10}
6.863 – 6.844	1.796 x10^{-10}
3.846 – 3.801	1.815 x10^{-10}
3.732 – 3.709	1.757 x10^{-10}
3.583 – 3.540	1.757 x10^{-10}

3.276 – 3.237	1.696 x10^{-10}
3.224 – 3.185	1.800 x10^{-10}

Average – 1.753 x10^{-10} m^2 s^{-1}

Standard Deviation – 0.048

2D DOSY spectra of S-MGB **4** and S-MGB **10** overlay

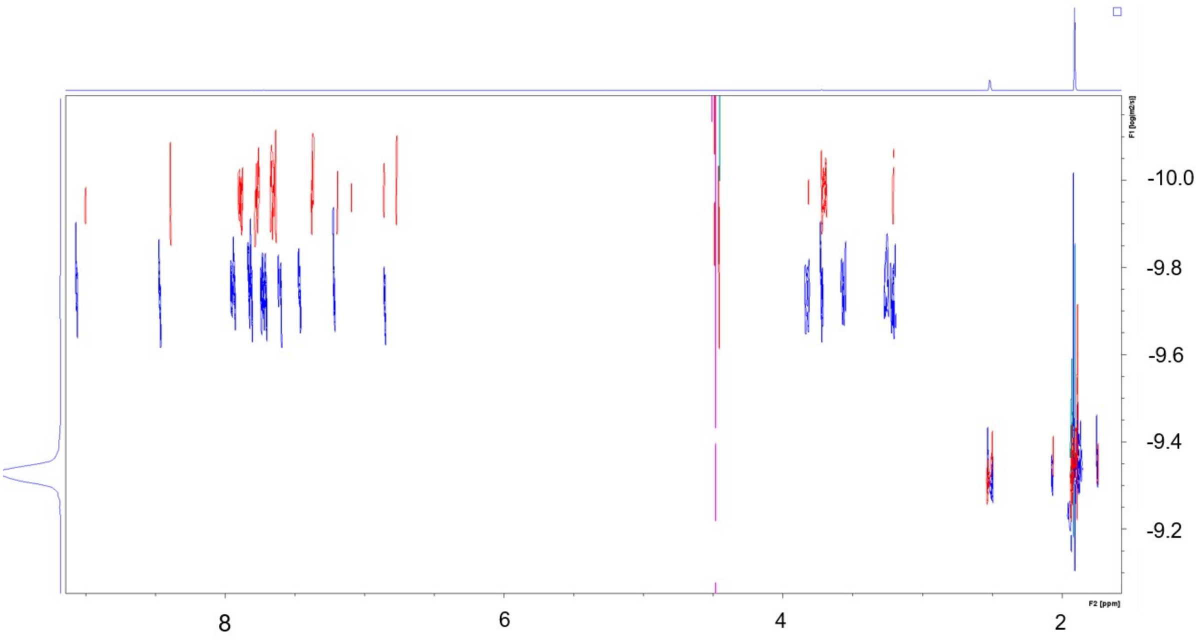

Figure S15. 2D DOSY NMR of both **4** (blue) and **10** (red) overlaid. Solvent system 50 % DMSO-d_6: 50 % 0.1M pH5 sodium acetate buffer.
